# Genetic regulation of fatty acid content in adipose tissue

**DOI:** 10.1016/j.ajhg.2025.12.008

**Published:** 2026-01-14

**Authors:** Xinyu Yan, Amy L. Roberts, Julia S. El-Sayed Moustafa, Sergio Villicaña, Maryam Al-Hilal, Max Tomlinson, Cristina Menni, Thomas A.B. Sanders, Maxim B. Freidin, Jordana T. Bell, Kerrin S. Small

**Affiliations:** 1 Department of Twin Research and Genetic Epidemiology, King’s College London, SE1 7EH London, UK; 2 Department of Medical and Molecular Genetics, King’s College London, SE1 9RT London, UK; 3 Department of Nutritional Sciences, King’s College London, SE1 9NH London, UK; 4 Health and Vital Statistics Division, National Center for Health Information, Ministry of Health, Sulaibkhat 13001, Kuwait; 5 Department of Pathophysiology and Transplantation, Università Degli Studi di Milano, 20122 Milan, Italy; 6 Fondazione IRCCS Cà Granda Ospedale Maggiore Policlinico, Angelo Bianchi Bonomi Hemophilia and Thrombosis Center, 20122 Milan, Italy

**Keywords:** fatty acid, adipose tissue, genome-wide association study, polygenic score

## Abstract

Fatty acids are important as structural components, energy sources, and signaling mediators. While studies have extensively explored genetic regulation of fatty acids in serum and other bodily fluids, their regulation within adipose tissue, a crucial regulator of cardiovascular and metabolic health, remains unclear. Here, we investigated the genetic regulation of 18 fatty acids in subcutaneous adipose tissue from 569 female twins from TwinsUK. Using twin models, the heritability of fatty acids ranged from 5% to 59%, indicating a substantial genetic regulation of fatty acid levels within adipose tissue, which was also tissue specific in many cases. Genome-wide association studies identified 10 significant loci, in *SCD*, *ADAMTSL1*, *ZBTB41*, *SNTB1*, *EXOC6B*, *ACSL3*, *LINC02055*, *MKRN2/TSEN2*, *FADS1*, and *HAPLN* across 13 fatty acids or fatty acid product-to-precursor ratios. Using adipose gene expression and methylation, which were concurrently measured in these samples, we detected five fatty acid-associated signals that colocalized with expression quantitative trait locus (eQTL) and methylation quantitative trait locus (meQTL) signals, highlighting fatty acids that are regulated by molecular processes within adipose tissue. We explored links between polygenic scores of common metabolic traits and adipose fatty acid levels and identified associations between polygenic scores of BMI, body-fat distribution, and triglycerides and several fatty acids, indicating these risk scores impact local adipose tissue content. Overall, our results identified local genetic regulation of fatty acids within adipose tissue and highlighted their links with renal and cardio-metabolic health.

## Teaser

Fatty acids function as energy sources, structural components, and signaling mediators. To investigate their genetic regulation across tissues, we profiled fatty acid content in adipose tissue biopsies from 569 twins. We identify heritable fatty acids and genetic loci regulating fatty acid content (and conversion ratios) within this key cardio-metabolic tissue.

## Introduction

Adipose tissue is a major endocrine organ and a crucial regulator of cardiovascular health. Adipose tissue secretes a range of bioactive products such as adipokines, leptin, adiponectin, and other hormones through endocrine and paracrine mechanisms. It lies at the heart of a regulatory network influencing energy balance, glycolipid metabolism, cardio-metabolic health, and immune response.^1^^,^^2^^,^^3^ Adipose tissue is the largest fat depot, storing and releasing fatty acids as needed.^4^ Fatty acids are monocarboxylic acids with a long hydrocarbon linear chain, saturated or unsaturated acids, with an even number of carbon atoms. Fatty acids function as energy sources, structural components, and signaling mediators, and they contribute to a range of molecular processes critical to common diseases, including neurological and cardiovascular disorders, type 2 diabetes (T2D), and cancers.^2^^,^^5^ Understanding the genetic regulation of fatty acids in adipose tissue may reveal the genetic and molecular mechanisms underlying risk of cardio-metabolic and other disorders.

Due to the fundamental role of circulating fatty acids and accessibility of blood samples, a plethora of studies have investigated the genetic regulation of circulating fatty acids, usually profiled in serum or plasma. Circulating fatty acids are under strong genetic regulation, with heritability estimates ranging from 10% to 48%.^6^^,^^7^ To date, genome-wide association studies (GWASs) have identified thousands of single-nucleotide polymorphisms (SNPs) associated with fatty acid metabolism in plasma or serum^8^^,^^9^^,^^10^^,^^11^ (GWAS catalog, https://www.ebi.ac.uk/gwas/), and several recent studies have conducted GWASs of fatty acids in bodily fluids such as feces,^12^ urine,^13^ and saliva.^14^^,^^15^ In contrast, limited studies^16^^,^^17^ have explored genetic associations of adipose tissue fatty acids, but these are limited to candidate SNP studies or GWASs limited to estimates of three fatty acid desaturases activity (delta-6-desaturase [D5D], delta-6-desaturase [D6D], and stearoyl-coenzyme A (CoA) desaturase-1 [SCD]),^17^ and there are currently no publicly accessible GWAS summary statistics of adipose fatty acid levels. Here, we investigate the genetic regulation of adipose tissue fatty acids levels by profiling 18 adipose fatty acids in subcutaneous adipose tissue biopsies from 569 females from the TwinsUK cohort. We utilize classic twin models to estimate heritability of these fatty acids and their ratios (fatty acid ratios can serve as proxies of enzymatic activity) and compare them with matched serum data from the same individuals to estimate tissue specificity of heritability. We conducted GWASs for the 18 fatty acids and 15 heritable fatty acid ratios and identified 10 genome-wide significant association signals. To elucidate the underlying molecular mechanisms at the GWAS loci, we integrated the fatty acid levels with gene expression and DNA methylation data. Finally, to define the link between genetic regulation of cardio-metabolic traits and adipose fatty acids, we explored the association between polygenic scores of cardio-metabolic traits and fatty acid levels.

## Material and methods

### Participants and samples

**Figure 1:**
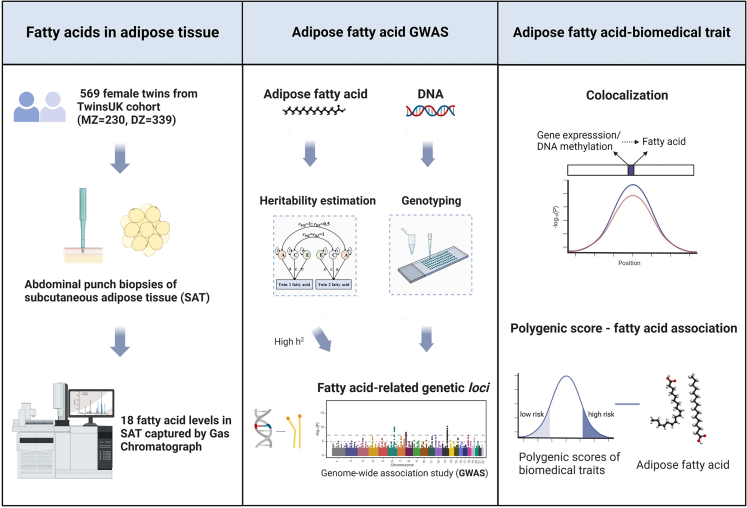
Study design of genetic regulation of fatty acid content in adipose tissue

The study included 569 female twins from the TwinsUK population cohort^18^ with available adipose fatty acid data. The study cohort consisted of 243 twin pairs (105 monozygotic [MZ] and 138 dizygotic [DZ] twin pairs) and 83 singletons, all of European ancestry, with a mean age of 59 (SD 9) years old and a mean body mass index (BMI) of 26.53 (SD 4.67) kg/m^2^. Punch biopsies were taken from a sunlight-protected area of the stomach adjacent and inferior to the umbilicus and immediately divided into aliquots and snap frozen, with one aliquot allocated to fatty acid profiling, one for RNA extraction, and one for DNA extraction and methylation profiling. All samples and information were collected with written and signed informed consent, including consent to publish within the TwinsUK study. TwinsUK has received ethical approval associated with TwinsUK Biobank (19/NW/0187), TwinsUK (EC04/015), or Healthy Aging Twin Study (HATS) (07/H0802/84) studies from NHS Research Ethics Service Committees London – Westminster. The study design is presented in Figure 1.

### Fatty acid profiling

#### Adipose fatty acid profiling

Fatty acid methyl esters were separated on an Agilent 7890 Gas Chromatograph (Agilent Technologies) fitted with a 60-m capillary column BPX70 with hydrogen as carrier gas and a flame ionization detector. The identities of fatty acids for which standards were not available were estimated using plots of the retention times as described elsewhere^19^ to obtain the equivalent chain lengths and n-9/n-6/n-3 ratios. Quality control was maintained by running a matrix standard of the sample of adipose tissue fatty acid methyl esters with each run. Chromatograms were evaluated using ChemStation software (version 8.04), and the output was exported via a visual basic application into Excel for data analysis. Detailed information was described elsewhere.^20^ A total of 18 types of fatty acids were quantified as proportions to the total fatty acid in 569 twins. Adipose fatty acids were summed up to obtain three classes of fatty acid values for each participant, saturated fatty acid (SFA), monounsaturated fatty acid (MUFA), and polyunsaturated fatty acid (PUFA). Adipose fatty acid ratios were calculated as product-to-precursor ratios of fatty acids to reflect the conversion rate of fatty acids.^21^ Rank-based inverse normal transformation was applied to the raw adipose fatty acid and fatty acid ratio values before analyses.

#### Serum metabolite profiling

Fatty acids in serum were extracted from a larger TwinsUK Metabolon metabolite dataset (*n* = 6,055), which includes 15 of the fatty acids also profiled in adipose tissue. The non-targeted metabolomics analysis was performed at Metabolon (Durham, NC, USA) on an ultra-high-performance liquid chromatography-tandem mass spectrometry (UPLC-MS/MS) instrument. Methods to process samples and data were described by Long et al.^7^ Free fatty acid levels were rank-based inverse normalized before analyzing. Date-matched serum Metabolon profiles were available for 471 of the twins with adipose fatty acid quantifications available.

### Genotyping and imputation

SNPs were genotyped using a combination of Illumina HumanHap300 (317k), HumanHap610Q (610k), 1M-Duo, and 1.2M-Duo (1M) arrays. Autosomal genotype data were imputed using the Haplotype Reference Consortium (HRC) v1.1 panel on the Michigan Imputation server (human reference build hg37). Quality-control measures were applied, including Hardy-Weinberg equilibrium (HWE) *p* > 10^−6^, minimum genotyping success rate >0.95, imputation quality R^2^ > 0.5, and minimum minor allele frequency (MAF) > 0.01, resulting in 7,656,749 retained autosomal SNPs. Human reference hg38 coordinates were generated using LiftOver. A total of 538 individuals had both autosomal genotype and adipose fatty acid data available.

Chromosome X genotypes were derived from whole-genome sequencing as part of the UK10K project. Briefly, low-coverage whole-genome sequencing data were generated using Illumina HiSeq 2000 and reads were mapped to human reference build hg37. Quality-control measures were applied, including a 99.50 truth sensitivity threshold for variant filtering, HWE *p* > 10^−6^, and MAF > 0.01, resulting in 331,002 retained chromosome X SNPs. Human reference hg38 coordinates were generated using LiftOver. A total of 352 individuals had both chromosome X genotype and adipose fatty acid data.

### Heritability estimates

Heritability of fatty acids in adipose tissue and serum in the TwinsUK cohort were estimated as proportions of total variance explained by additive genetic effects using the ACE twin model, which modeled trait variance as a function of additive genetics (A), common environment (C), and unique environment effects (E) using the structural equation modeling software OpenMx.^22^ 243 complete twin pairs (486 twins) were used to estimate adipose fatty acid heritabilities, and 195 complete twin pairs (390 twins) were used to estimate serum fatty acid heritabilities. In the model to estimate the heritability of fatty acids in adipose tissue, age was included as a covariate, followed by sensitivity analyses where (1) age and cell types (adipocyte, macrophage, and microvascular endothelial cell [MVEC])^23^; (2) age and BMI; and (3) age, BMI, and cell types were included as covariates. Only age was adjusted for when estimating the heritability of serum fatty acids.

### Discovery genome-wide association analysis

Discovery GWASs were performed across 18 individual fatty acids, three fatty acid sums (SFA, MUFA, and PUFA), and 15 heritable (h^2^ ≥ 0.15) fatty acid ratios of product-to-precursor fatty acids. The Wald test implemented in GEMMA V0.98.1^24^ was utilized to assess the significance of the associations, accounting for family relatedness using a sample kinship matrix, using linear mixed models adjusting for age. Three thresholds were applied: (1) genome-wide significance, *p*_G_ < 5 × 10^−8^ (the golden standard for GWAS, accounting for multiple testing in GWAS where ∼10^6^ SNPs are tested simultaneously); (2) Bonferroni-corrected study-wide genome-wide significance, *p*_B_ < 2.8 × 10^−9^ (5 × 10^−8^/18, where 18 represents the number of independent effective tests performed; and (3) suggestive significance, *p*_S_ < 10^−6^ (less strict threshold for exploratory analysis). In the second threshold (*p*_B_), the study-wide threshold was corrected for the number of independent effective tests (*N* = 18) to account for the high degree of inter-correlation among the fatty acids and ratios. The number of independent effective tests was calculated by applying the meff function in the R package poolr to the correlation matrix of the 38 fatty acids and ratios used in GWASs. To test whether any of the associated loci had multiple distinct association signals, conditional analysis was performed by including the lead variant (*p* < 5 × 10^−8^) as a covariate. The locus-wide significance threshold was an arbitrary set of *p* < 10^−4^ to identify secondary association signals within ±100 kb of the primary GWAS signal. This threshold accounts for the reduced multiple testing burden due to local linkage disequilibrium (LD) structure and balances sensitivity for detecting true secondary signals while controlling multiple testing within a defined locus. We also performed sensitivity analyses adjusted for (1) age and cell-type composition; (2) age and BMI; and (3) age, BMI, and cell-type composition in the GWAS model, in order to identify the impact of these covariates on the analyses. The variance explained by the lead variant was calculated using the formula 2 × EAF × (1 − EAF) × Beta^2^/Var(Y), in which EAF is the effect allele frequency, Beta is the effect estimate of the effect allele, and Var(Y) is the variance of the phenotype Y.^25^ Genomic inflation factor (lambda genomic control) was calculated using Devlin and Roeder’s formula.^26^ Manhattan plots and QQ plots were produced by the R package qqman, and Locus zoom plots were generated using the open-source software LocusZoom.js.^27^

### Replication genome-wide association analysis

#### Blood metabolites studies

(1) TwinsUK matched serum dataset and TwinsUK-Cooperative Health Research in the Region of Augsburg (KORA). The serum-metabolite profiling method was mentioned as above. We tested seven SNP-fatty acid association pairs across five available fatty acids in serum-metabolite datasets (*n* = 448). Due to technical differences in methods used in the serum metabolite profiling, quantification of SFA was not available in serum. Meta-analysis combining TwinsUK and KORA (Augsburg) participants’ metabolites GWAS results were further included (*n* = 7,824).^9^ (2) European Prospective Investigation of Cancer (EPIC)-Norfolk and INTERVAL study. Blood metabolites were measured using the untargeted platform Metabolon in 19,994 individuals from UK-based studies, EPIC-Norfolk and INTERVAL, and GWASs of metabolites were performed.^10^ (3) Canadian Longitudinal Study of Aging (CLSA). Blood metabolites were quantified using UPLC-MS/MS platform in 8,299 individuals from a Canada-based study, CLSA, and GWASs were performed across metabolites.^11^

### RNA-seq and DNA methylation

#### RNA-seq

RNA sequencing (RNA-seq) was performed to measure gene expression levels in TwinsUK participants in adipose tissue (*n*= 765), skin (*n* = 706), lymphoblastoid cell lines (LCLs) (*n* = 804), and whole blood (*n* = 389), as previously reported.^28^ RNA-seq reads were aligned to the GRCh37 (hg19) reference genome using STAR v2.4.0.1^29^ and then quality control was conducted.^30^^,^^31^ Gene-level counts were quantified using the quan function from QTLtools^32^ and Gencode v19.^33^ RNA-seq gene expression data were filtered to retain genes with five or more counts per million (CPM) in ≥25% of individuals. Read counts were adjusted for each gene for the trimmed mean of M values (TMM)^34^ and were rank-based inverse normalized.

#### DNA methylation

The DNA was bisulfite-converted using the EZ DNA Methylation Kit (Zymo Research). The DNA methylation profiles of adipose tissue were conducted in 588 twins using Illumina Infinium HumanMethylation450 BeadChip (450K array)^35^ to obtain β values, which represent the proportion of methylated probes at a specific CpG site.^36^ The raw methylation data were processed, including background correction, dye-bias adjustment, signal normalization, and the estimation of adjusted β values, as described previously.^37^ DNA methylation levels of CpG sites were rank-based inverse normalized prior to association analysis using linear models.

#### Smoking status

As 76 individuals were missing self-reported smoking status, predicted smoking status was estimated for all individuals using DNA methylation biomarkers of smoking, as described in Tsai et al.^38^

#### QTL analysis

(1) Expression quantitative trait loci (eQTLs). Association between SNP and gene expression level was performed using a linear mixed-effect regression model, adjusting for BMI, family, zygosity, SNP genotyping chip, and estimation of expression residuals (PEER) factors, following the methods described previously.^39^^,^^40^ Conditional eQTLs were carried out if the *p* value of the top SNP was lower than 10^−5^ by including the genotype of the top SNP as an additional covariate. (2) eQTL and splicing quantitative trait loci (sQTL) across tissues. We used multi-tissue eQTL and sQTL summary statistics in ∼706 individuals, which were generated from the GTEx project (v8, European ancestry).^41^ (3) Methylation quantitative trait loci (meQTLs). Association between SNP and DNA methylation level was performed adjusting for technical covariates, age, predicted smoking, family relatedness, genetic PCs, and non-genetic DNA methylation PCs from DNA methylation, as described previously.^42^

### QTL-fatty acid colocalization analysis

#### eQTL/meQTL-fatty acid colocalization

To test whether the GWAS lead variant was colocalized with the lead variant of gene expression or DNA methylation level, we filtered pairwise LD r^2^ between the GWAS lead variant and eQTL/meQTL lead variant within 1 Mb greater than 0.6 of European ancestry population using the LDpair module on NIH LDlink (https://ldlink.nci.nih.gov/?tab=apiaccess). Next, to formally test whether gene expression/DNA methylation (trait 1) and adipose fatty acid (trait 2) share a common genetic causal variant in a given region (hypothesis 4 [H4]), we ran GWAS-eQTL/meQTL colocalization analysis within 500 kb using the coloc v5.1.0.1 R package.^43^ Sensitivity analysis was performed using the “sensitivity” function in the coloc package, *post hoc*, to determine the range of prior probabilities for which a conclusion is still supported. We defined the variants as colocalized when the posterior probability of colocalized signal (PP.H4) was greater than 0.75.^43^ We also colocalized GWAS secondary variant with eQTL/meQTL lead variant and colocalized GWAS lead variant with eQTL/meQTL secondary variant using the same pipeline.

#### Cell-type composition estimates

Adipose tissue is composed of multiple cell types, with the most common being adipocyte, endothelial, and immune cells. We estimated adipose tissue cell-type proportion from RNA-seq data in TwinsUK, including adipocytes, macrovascular endothelial cells, and macrophages, as previously described.^23^

#### Fatty acid gene expression/DNA methylation association analysis

We assessed the association between adipose gene expression and fatty acids levels in 525 twins with both measures available, adjusting for technical covariates, age, BMI, and family relatedness as covariates by linear mixed model using the lme4 R package.^44^ Then we tested the association between adipose DNA methylation and fatty acids in 394 twins with both measures available, adjusting for technical covariates, age, predicted smoking, family relatedness, and non-genetic DNA methylation PCs. Gene expression or DNA methylation was treated as a continuous independent fixed effect, and each adipose fatty acid was treated as a continuous dependent variable.

### Fatty acid-kidney trait colocalization analysis

To test whether n-6 PUFAs in adipose tissue shared a common genetic causal variant with kidney traits, we integrated GWASs of TwinsUK adipose fatty acids with published GWASs of kidney trait^45^ using the same pipeline. Both arachidonic acid level and the ratio of arachidonic acid to linoleic acid were included. Three kidney traits were included in the analysis: eGFR, serum creatinine, and cystatin C.

### Polygenic score analysis

We constructed polygenic scores (PGSs) for 13 common metabolic traits: abdominal subcutaneous adipose tissue volumes adjusted for BMI (ASATadjBMI), visceral adipose tissue volumes adjusted for BMI (VATadjBMI), gluteofemoral adipose tissue volumes adjusted for BMI (GFATadjBMI), BMI, waist-to-hip ratio adjusted for BMI (WHRadjBMI), type 1 diabetes (T1D), T2D, coronary artery disease (CAD), hypertension, high-density lipoprotein cholesterol (HDL), low-density lipoprotein cholesterol (LDL), log-transformed total triglycerides (TGs), and total cholesterol (TC).^46^^,^^47^^,^^48^^,^^49^^,^^50^^,^^51^^,^^52^ PGSs were calculated for 6,847 participants using the linear scoring method implemented in PLINK 1.9 “--score” function (Table S1). Then, we validated PGSs in TwinsUK where PGSs showed broadly strong associations with corresponding traits (Figure S1; Table S2 and supplemental material and methods). Finally, we tested the association between PGSs and fatty acid levels and summary levels of fatty acids (SFA, MUFA, and PUFA) for 533 twins with genotypes and fatty acid quantifications available, adjusting for age, cell types, and relatedness excluding participants with T1D, T2D, cardiovascular disease (CVD), or hypertension. To overcome the multiple-test burden, *p* values were Bonferroni corrected using the effective number of fatty acids (*N*_eff_ = 13) for each PGS.

## Results

### Fatty acid characteristics in adipose tissue

We measured 18 types of fatty acids among 569 female twins (243 twin pairs and 83 singletons) in subcutaneous adipose tissue (Figure 1), including six SFAs, four MUFAs, and eight PUFAs including four n-6 family and four n-3 family PUFAs. Summary level values of SFA, MUFA, and PUFA were calculated from individual measurements. The characteristics of adipose fatty acid proportions in our study are shown in Table 1. Overall, the values are in line with expectations from previous studies of adipose tissue,^53^^,^^54^ including high median levels of palmitic acid, oleic acid, and linoleic acid, which together account for ∼78% of fatty acids within adipose tissue.

**Table 1:** Characteristics and heritability of fatty acid in adipose tissue

**Adipose fatty acid**	**Common name**	***N***	**Median**	**Mean**	**Variance**	**Adipose fatty acid h**^**2**^	**Serum fatty acid h**^**2**^
SFA	saturated fatty acid	543	25.62	25.74	8.28	0.28	–
C10:0	capric acid	546	0.25	0.33	0.13	0	0.27
C12:0	lauric acid	566	0.34	0.61	0.53	0.09	0.05
C14:0	myristic acid	567	2.17	2.03	0.75	0	0.44
C16:0	palmitic acid	569	19.28	19.24	4.23	0.30	0.34
C18:0	stearic acid	569	3.10	3.21	0.89	0.51	0.33
C20:0	arachidic acid	563	0.35	0.36	0.03	0	–
MUFA	monounsaturated fatty acid	502	53.25	52.97	10.68	0.12	–
C16:1n-7	palmitoleic acid	567	5.75	5.79	2.77	0.05	0.42
C18:1n-7	vaccenic acid	504	0.29	0.88	0.88	0.04	–
C18:1n-9	oleic acid	569	46.77	46.55	9.29	0	0.40
C18:1trans	elaidic acid, *trans*-vaccenic acid	497	0.51	0.53	0.09	0.43	–
PUFA	polyunsaturated fatty acid	557	14.79	15.03	6.06	0.31	–
C18:3n-3	α-linolenic acid (ALA)	564	0.85	0.85	0.05	0.50	0.21
C20:5n-3	EPA	561	0.17	0.22	0.02	0	0.49
C22:5n-3	docosapentaenoic acid (DPA)	561	0.36	0.39	0.03	0.29	0.22
C22:6n-3	DHA	561	0.31	0.36	0.05	0	0.31
C18:2n-6	linoleic acid (LA)	568	11.92	12.05	5.61	0.42	0.31
C20:3n-6	dihomo-γ-linolenic acid (DGLA)	563	0.27	0.32	0.03	0.33	0.35
C20:4n-6	arachidonic acid	563	0.54	0.56	0.03	0.59	0.28
C22:4n-6	adrenic acid	561	0.21	0.26	0.03	0.23	0.20

Median, mean, and variance showed the distribution of the proportion of each type of fatty acid in the subcutaneous adipose biopsy; *N*, sample size; the heritability (h^2^) was estimated by the ACE model, adjusted for age among 486 twins (210 MZ and 276 DZ) and 390 twins (214 DZ and 176 MZ) for adipose and serum fatty acid, respectively.

**Figure 2:**
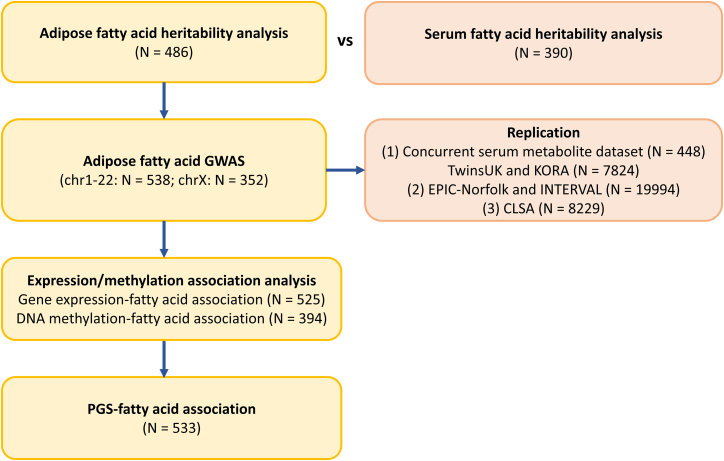
Flowchart of the study including sample sizes in each analysis

Throughout this study, the adipose fatty acid levels were integrated with pre-existing data from the TwinsUK cohort across a range of analyses. The sample size in different analyses varied depending on the availability of overlapping pre-existing data. A flowchart of the study design, including the sample size used in each analysis, is provided in Figure 2, and the overlap of individuals across all datasets is shown in Figure S2.

### The heritability of fatty acids is highly variable and tissue specific

#### Adipose fatty acid

To understand how genetic variation contributes to adipose fatty acid contents, we utilized classic twin models to calculate the heritability of 18 individual fatty acids and the summary values of SFAs, MUFAs, and PUFAs (Figure 1). We observed a broad range of heritability (0%–59%, Table 1), with the highest heritability observed for arachidonic acid. Overall, individual PUFAs showed consistent moderate to high heritability (h^2^ = 23%–59%), with the exception of eicosapentaenoic acid (EPA) and docosahexaenoic acid (DHA) (h^2^ ∼ 0%). Conversely, only two of six SFAs (palmitic acid and stearic acid) were highly heritable (h^2^ = 30% and 51%, respectively), while the remaining SFAs and all three MUFAs had low heritability (h^2^ < 10%).

Next, we calculated the heritability of 41 product-to-precursor ratios of fatty acids. Once more, we observed a broad range of heritability (h^2^ = 0%–54%) (Table 2). Fifteen product-to-precursor ratios were heritable (h^2^ ≥ 15%), and the mean heritability of the product-to-precursor ratios (h^2^ = 29%) was greater than the mean heritability of the individual-level fatty acids described above (Table 1), suggesting genetic factors contribute more to the enzymatic activity and conversion of fatty acids than their absolute levels.

**Table 2:** Heritability of fatty acid product-precursor ratios in adipose tissue

**Adipose fatty acid ratio**	**h**^2^
C16:0/C12:0 (palmitic acid/lauric acid)	0.17
C18:0/C12:0 (stearic acid/lauric acid)	0.17
C18:0/C16:0 (stearic acid/palmitic acid)	0.47
C20:0/C18:0 (arachidic acid/stearic acid)	0.18
C16:1n-7/C16:0 (palmitoleic acid/palmitic acid)	0.19
C18:1n-9/C18:0 (oleic acid/stearic acid)	0.47
C18:1n-7/C16:1n-7 (vaccenic acid/palmitoleic acid)	0.22
C20:3n-6/C18:2n-6 (dihomo-γ-linolenic acid/linoleic acid)	0.46
C20:4n-6/C18:2n-6 (arachidonic acid/linoleic acid)	0.54
C20:4n-6/C20:3n-6 (arachidonic acid/dihomo-γ-linolenic acid)	0.53
C22:4n-6/C18:2n-6 (adrenic acid/linoleic acid)	0.31
C22:4n-6/C20:3n-6 (adrenic acid/dihomo-γ-linolenic acid)	0.44
C22:4n-6/C20:4n-6 (adrenic acid/arachidonic acid)	0.23
C22:5n-3/C18:3n-3 (docosapentaenoic acid/α-linolenic acid)	0.15
C22:6n-3/C22:5n-3 (DHA/docosapentaenoic acid)	0.28

The heritability (h^2^) was estimated by the ACE model, adjusted for age among 486 twins (210 MZ and 276 DZ).

#### Serum fatty acid

To compare the relative contribution of genetic regulation of fatty acids in different tissues, we investigated the heritability of 15 fatty acid levels in serum samples from a subset of the TwinsUK individuals (195 pairs of twins), which were profiled using the Metabolon platform on the same day as their adipose biopsy. Consistent with our adipose results, fatty acids in serum showed a range of heritabilities (h^2^ = 5%–49%) (Table 1). While we saw similarity in the heritability estimates across serum and adipose tissue for some fatty acids (e.g., palmitic acid, docosapentaenoic acid, linoleic acid, dihomo-γ-linolenic acid and adrenic acid), others showed great discrepancies in heritability across tissues (Figure S3). For example, myristic acid was highly heritable (h^2^ = 44%) in serum yet was not heritable in adipose tissue (h^2^ = 0%), whereas both arachidonic acid and α-linolenic acid showed substantially higher heritability in adipose than serum (Table 1). Notably, the most common fatty acid in adipose, oleic acid, showed no heritability in adipose but high heritability in serum (h^2^ = 40%). Overall, these results indicated substantial genetic regulation of fatty acids in both adipose tissue and serum, with some important tissue-specific effects. We saw no difference in heritability results when including BMI and/or cell-type composition as covariates (Tables S3 and S4).

### Identification of genetic variants associated with adipose fatty acids

**Figure 3:**
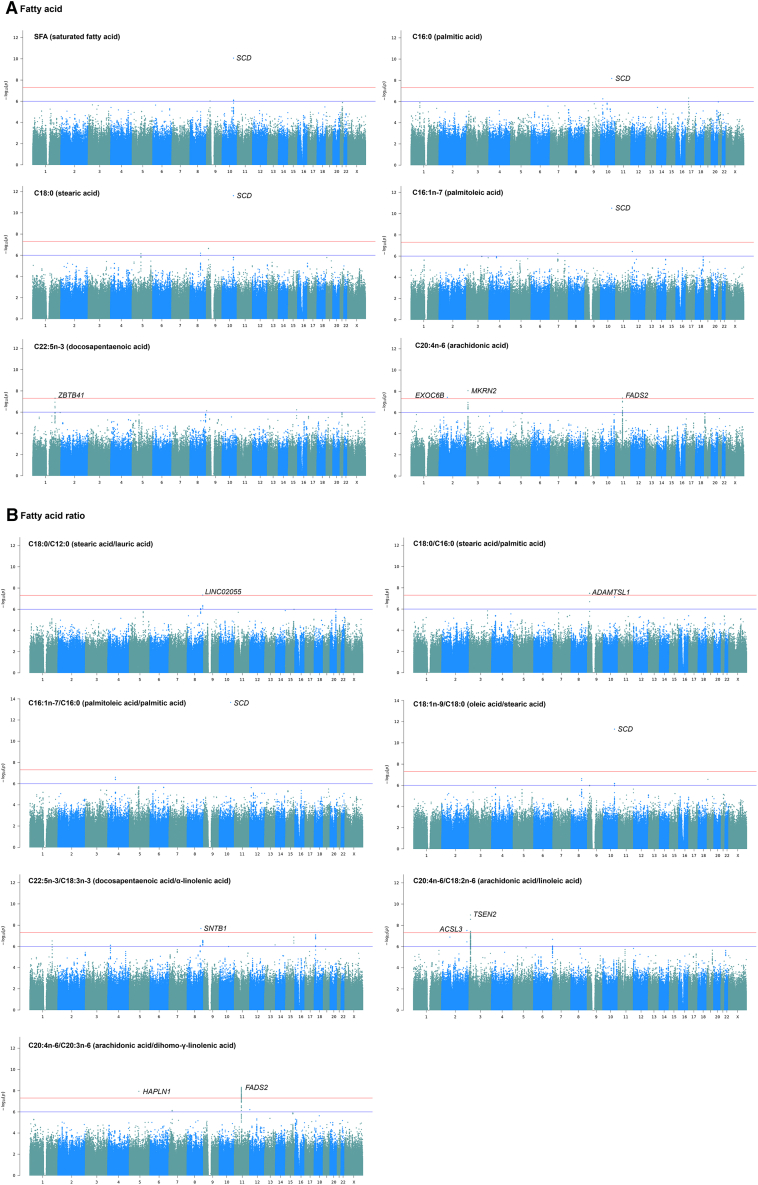
Manhattan plots of GWASs on fatty acids in adipose tissue The *x* axis indicates chromosome 1–22 and chromosome X, the *y* axis indicates minus log10-transformed GWAS *p* value, the red horizontal line demarcates the genome-wide significance threshold of *p*_*G*_ = 5 × 10^−8^, the genome-wide significant loci are marked and annotated to the nearest genes, and the blue line indicates suggestive significance threshold of *p*_*s*_ = 10^−6^.

In order to identify genetic loci associated with adipose fatty acid levels, we performed GWASs of 18 individual fatty acids, three fatty acid sums (SFA, MUFA, and PUFA) and 15 ratios of product-to-precursor fatty acids (h^2^ ≥ 0.15) (Figure 1). We identified 10 independent genome-wide significant associated loci (*p*_*G*_ < 5 × 10^−8^) across six fatty acids and seven fatty acid ratios (Table 3; Figures 3, S4, and S5), eight of which were novel discoveries, which do not overlap with any locus previously reported as genome-wide significant for fatty acids in adipose tissue. Two of 10 associated loci (*SCD* [MIM: 604031] locus, lead variant rs603424; and 3p25.2 locus, lead variant rs6768977) were significant at a study-wide multiple testing corrected threshold accounting for the number of independent fatty acids (*p*_*B*_ < 2.8 × 10^−9^), among which 3p25.2 locus (rs6768977) was a novel finding. The *SCD* locus (rs603424) was associated with SFA, palmitic acid, stearic acid, palmitoleic acid, the ratio of palmitoleic acid/palmitic acid and the ratio of oleic acid/stearic acid. The lead variant explained 7.0%–11.2% of the variance in SFA and MUFA. Variants at the *FADS* cluster have been widely reported to regulate the desaturation of PUFAs; in this study, we identified associations to arachidonic acid and the ratio of arachidonic acid/dihomo-γ-linolenic acid. The lead variants at the *FADS* locus, rs97384 and rs174544, were located in the protein-coding gene *FADS2* (MIM: 606149), close to *FADS1*/*FEN1*/*TMEM258* (MIM: 606148/600393/617615), and explained 6.5% and 7.9% of the variance. By conditioning on the lead variant at the *FADS* locus (rs97384), we found four secondary variants at the *FADS* locus where the lead variant was rs61898565 (*p* = 6.3 × 10^−5^).

**Table 3:** Genome-wide significant loci of fatty acids in adipose tissue

**Adipose fatty acid**	**Variant**	**Chr**	**Pos**	**Nearest genes**	**Effect allele**	**Non-effect allele**	**EAF**	**Effect**	***SE***	***p***	**Variance explained by the variant (%)**
**Fatty acid**											

SFA	rs603424	10	100,315,722	*PKD2L1/SCD*	A	G	0.176	0.554	0.084	8.5E−11^a^	9.0
C16:0 (palmitic acid)	rs603424	10	100,315,722	*PKD2L1/SCD*	A	G	0.175	0.490	0.083	6.6E−09	7.0
C18:0 (stearic acid)	rs603424	10	100,315,722	*PKD2L1/SCD*	A	G	0.175	0.596	0.083	2.4E−12^a^	10.1
C16:1n-7 (palmitoleic acid)	rs603424	10	100,315,722	*PKD2L1/SCD*	A	G	0.175	−0.542	0.080	3.1E−11^a^	8.6
C22:5n-3 (docosapentaenoic acid)	rs114713999	1	197,160,467	*ZBTB41/ASPM*	A	G	0.012	1.521	0.275	4.8E−08^b^	5.5
C20:4n-6 (arachidonic acid)	rs17559539	2	72,348,460	*EXOC6B/CYP26B1*	C	A	0.044	−0.955	0.171	3.7E−08^b^	7.7
C20:4n-6 (arachidonic acid)	rs1542848	3	12,576,497	*MKRN2/RAF1/TSEN2/PPARG*	A	G	0.375	−0.379	0.065	8.7E−09^b^	6.8
C20:4n-6 (arachidonic acid)	rs97384	11	61,856,709	*FADS2/FADS1/FADS3/FEN1/TMEM258*	C	T	0.621	0.370	0.067	4.3E−08	6.5

**Fatty acid ratio**											

C18:0/C12:0 (stearic acid/lauric acid)	rs4460408	8	137,045,124	*LINC02055/RNU6-144P*	A	C	0.780	0.439	0.079	4.6E−08^b^	6.6
C18:0/C16:0 (stearic acid/palmitic acid)	rs776749	9	18,642,756	*ADAMTSL1/MIR3152*	A	G	0.535	0.372	0.066	3.3E−08^b^	6.8
C16:1n-7/C16:0 (palmitoleic acid/palmitic acid)	rs603424	10	100,315,722	*PKD2L1/SCD*	A	G	0.175	−0.624	0.079	2.2E−14^a^	11.2
C18:1n-9/C18:0 (oleic acid/stearic acid)	rs603424	10	100,315,722	*PKD2L1/SCD*	A	G	0.175	−0.587	0.083	5.1E−12^a^	9.8
C22:5n-3/C18:3n-3 (docosapentaenoic acid/α-linolenic acid)	rs79670584	8	120,585,786	*SNTB1/MTBP/MRPL13/COL14A1*	A	T	0.019	1.410	0.248	2.1E−08^b^	7.4
C20:4n-6/C18:2n-6 (arachidonic acid/linoleic acid)	rs795891	2	222,881,016	*ACSL3/MOGAT1*	G	A	0.820	0.480	0.085	3.1E−08^b^	6.9
C20:4n-6/C18:2n-6 (arachidonic acid/linoleic acid)	rs6768977	3	12,466,670	*TSEN2/PPARG/MKRN2/RAF1*	G	A	0.494	−0.379	0.061	1.1E−09^a^^,^^b^	7.2
C20:4n-6/C20:3n-6 (arachidonic acid/dihomo-γ-linolenic acid)	rs79680695	5	83,784,409	*HAPLN1/EDIL3*	G	C	0.050	0.854	0.147	1.2E−08^b^	7.0
C20:4n-6/C20:3n-6 (arachidonic acid/dihomo-γ-linolenic acid)	rs174544	11	61,800,281	*FADS2/FADS1/FEN1/TMEM258/FADS3*	A	C	0.307	−0.429	0.072	4.8E−09	7.9

Chr, chromosome; Pos, position (GRCh38/hg38); EAF, effect allele frequency; Effect, beta estimate of effect allele; SE, standard error; Variance explained, the variance explained by the lead variant. For each locus, only the lead variant was reported here.

a Bonferroni-corrected genome-wide significance with *P*_*B*_ < 2.8 × 10^−9^.

b Novel locus.

Six out of eight novel loci were associated with polyunsaturated fatty acids. The 3p25.2 locus (rs1542848 and rs6768977) was associated with arachidonic acid and the ratio of arachidonic acid/linoleic acid. The *CYP26B1* (MIM: 605207) locus (rs17559539) was associated with arachidonic acid. A total of 266 SNPs reached the suggestive threshold (*p*_*S*_ < 10^−6^) (Table S5), suggesting higher-powered GWASs may robustly identify further genetic associations.

In order to test the robustness of the approach, we have also performed sensitivity analyses by re-running all heritability estimates and GWASs, including the potential confounders, BMI, and/or cell types, as covariates. Overall, we see similar results across the analyses, with slight fluctuation of *p* values for some fatty acids (Tables S3, S4, and S6). Given the consistency in the results, all further analyses in the main text reference the GWAS results that do not adjust for BMI or cell-type composition.

### Replication and tissue specificity

We sought to replicate the genome-wide significant GWAS loci in both adipose tissue and serum/plasma datasets. The only published GWAS of fatty acids in adipose tissue investigated the association between estimates of adipose desaturase activity for D5D, D6D, and SCD as measured by fatty acid product-to-precursor ratios in buttock subcutaneous adipose tissue obtained from 783 men from the Uppsala Longitudinal Study of Adult Men (ULSAM) study.^17^ This study reported genome-wide significant associations of D5D (C20:4n-6/C20:3n-6 ratio) to the *FADS* locus and SCD (C16:1/C16:0 ratio) to the *SCD* locus, which are consistent with our results at these loci. A second study investigating candidate SNPs at the *FADS* locus with adipose tissue fatty acid levels in 173 European individuals from the Diet, Obesity and Genes Study (DiOGenes) also reported an association with D5D activity (C20:4n-6/C18:2n-6 ratio).^16^ However, the full summary statistics of the previous adipose fatty acid studies could not be made available, so we could not investigate further replication in adipose tissue. Therefore, we sought to replicate the GWAS results in serum metabolite datasets.

We first performed GWASs of matched serum metabolite dataset in TwinsUK where blood samples were collected at the same time as adipose tissue biopsies within the same group of participants (*n* = 448). We replicated the associations between *SCD* locus (rs603424) and *FADS* locus (rs97384) and three fatty acids (Table 4). We then sought to replicate the GWAS results in four larger cohorts, including a combined TwinsUK and KORA cohort (*n* = 7,824),^9^ a combined EPIC-Norfolk and INTERVAL cohort (*n* = 19,994),^10^ and CLSA cohort (*n* = 8,299).^11^ We replicated the association between *SCD* locus (rs603424) and stearic acid and palmitoleic acid, and the associations between *FADS* locus (rs97384 and its LD proxies, rs174548 and rs102275) and arachidonic acid level. Together, these results suggest the majority of testable loci are replicated and show shared metabolic pathways in both serum and adipose tissue.

**Table 4:** Replicated variant-fatty acid associations in other cohorts

**Fatty acid**	**Author**	**Cohort/Trial**	**Tissue**	**Chr**	**SNP**	**EA/OA**	**EAF**	**Effect**	**SE**	***p***	**N**
C16:0 (palmitic acid)	–	TwinsUK	serum	10	rs603424	A/G	0.165	0.183	0.085	3.2E−02	448
C18:0 (stearic acid)	–	TwinsUK	serum	10	rs603424	A/G	0.165	0.262	0.083	1.8E−03	448
C18:0 (stearic acid)	Shin et al.^9^	TwinsUK and KORA F4	blood	10	rs603424	A/G	0.180	0.023	0.002	6.1E−21	7,355
C16:1n-7 (palmitoleic acid)	Shin et al.^9^	TwinsUK and KORA F4	blood	10	rs603424	A/G	0.180	−0.037	0.005	1.3E−14	7,327
C16:1n-7 (palmitoleic acid)	Surendran et al.^10^	EPIC-Norfolk and INTERVAL	plasma	10	rs603424	A/G	0.180	−0.119	0.015	1.4E−14	14,263
C16:1n-7 (palmitoleic acid)	Chen et al.^11^	CLSA	blood	10	rs603424	A/G	0.176	−0.128	0.019	1.2E−11	8,245
C20:4n-6 (arachidonic acid)	–	TwinsUK	serum	11	rs97384	C/T	0.610	0.271	0.070	1.2E−04	448
C20:4n-6 (arachidonic acid)	Shin et al.^9^	TwinsUK and KORA F4	blood	11	rs174548^a^	C/G	0.700	0.049	0.003	1.4E−84	7,367
C20:4n-6 (arachidonic acid)	Surendran et al.^10^	EPIC-Norfolk and INTERVAL	plasma	11	rs97384	T/C	0.370	−0.352	0.012	1.1E−177	14,262
C20:4n-6 (arachidonic acid)	Chen et al.^11^	CLSA	blood	11	rs102275^a^	C/T	0.346	−0.314	0.016	1.4E−90	8,264
C20:4n-6/C20:3n-6 (arachidonic acid/dihomo-γ-linolenic acid)	Shin et al.^9^	TwinsUK and KORA F4	blood	11	rs174548^a^	C/G	0.700	0.071	0.002	1.53E−361	7,350

Chr, chromosome; EA/OA, effect/other allele; EAF, effect allele frequency; Effect, beta estimate of effect allele; SE, standard error; Direction, the association direction, Negative or Positive; N, sample size.

a SNP in replication cohort is in high LD with discovered SNP.

### GWAS signals are colocalized with eQTL and meQTL signals and highlight tissue specificity

**Figure 4:**
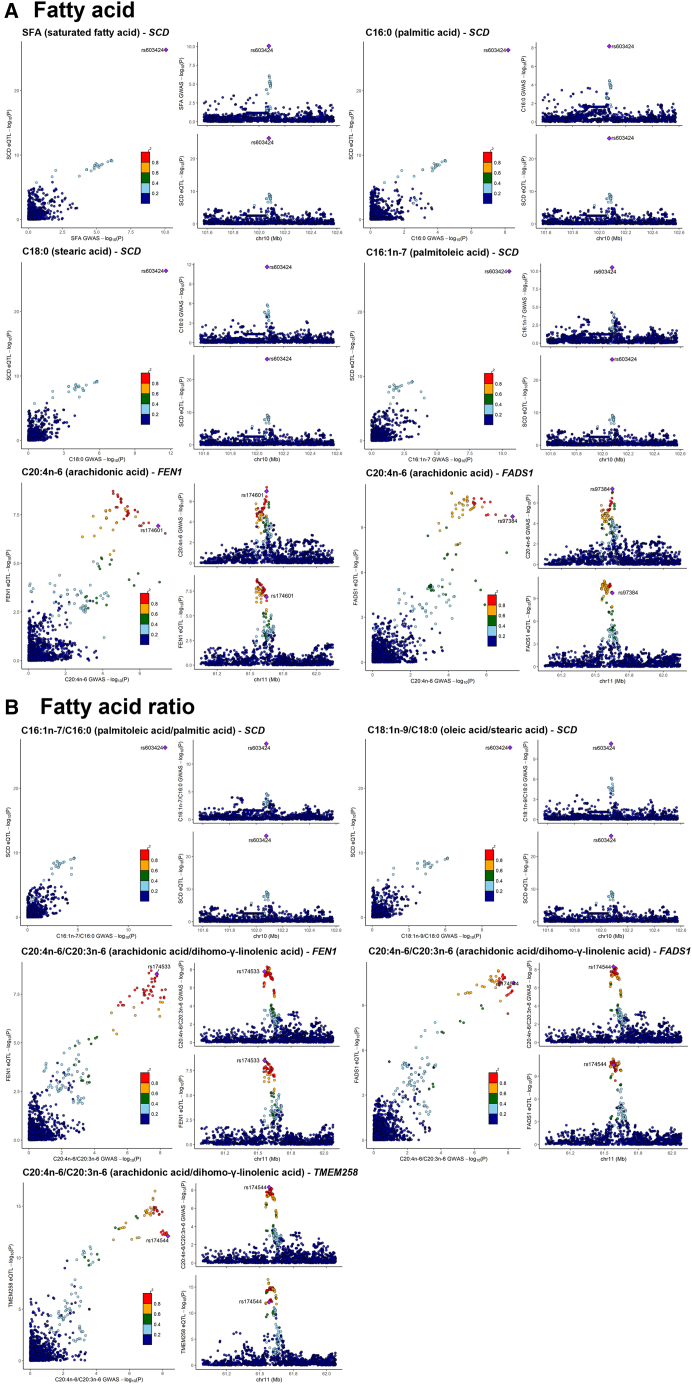
Colocalization of GWAS and eQTL signals The labeled SNP is the lead SNP for both GWAS and eQTL studies, and other SNPs are colored according to their LD r^2^ with the lead SNP.

**Figure 5:**
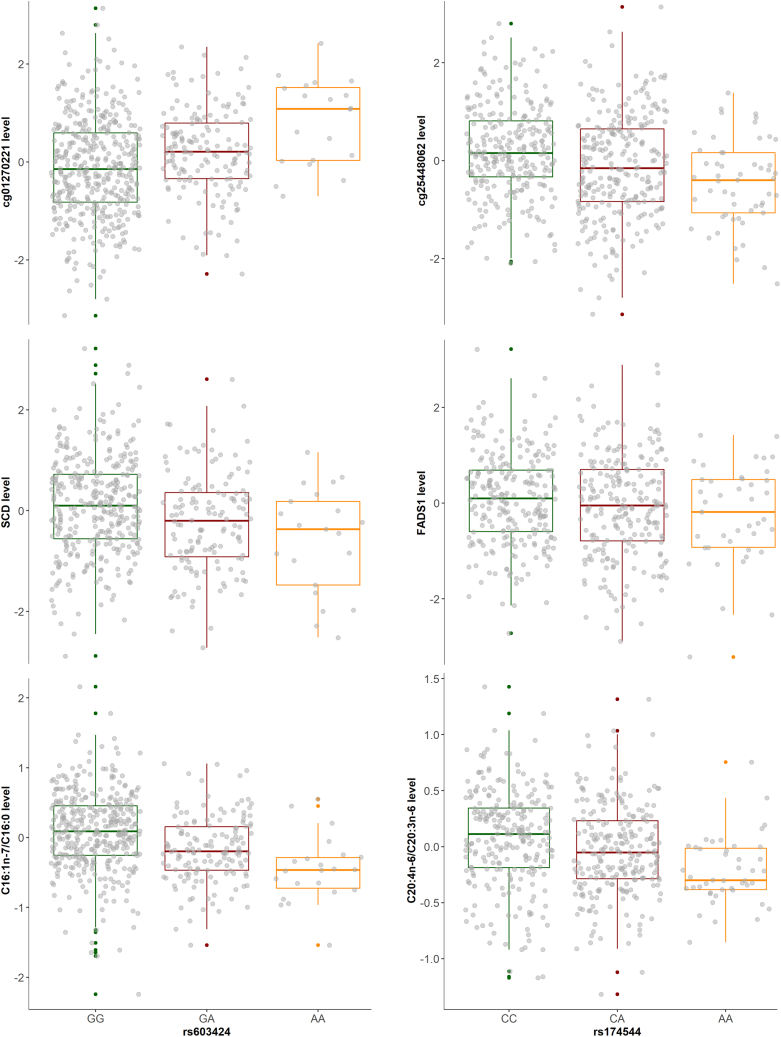
Boxplots of levels of DNA methylation, gene expression, and fatty acids on genotypes for colocalized GWAS-eQTL or meQTL signals The left plot shows the levels of cg01270221, *SCD* gene, and C16:1n-7/C16:0 (palmitoleic acid/palmitic acid) by the SNP rs603424 in adipose tissue; the right plot shows the levels of cg25448062, *FADS1* gene, and C20:4n-6/C20:3n-6 (arachidonic acid/dihomo-γ-linolenic acid) by the SNP rs174544 in adipose tissue. We take the most significant fatty acid or CpG associated with the same SNP as the examples here. We regress out age and family relatedness for fatty acid; regress out technical covariates, age, BMI, and family relatedness for gene expression; and regress out technical covariates, age, predicted smoking, family relatedness, genetic PCs, and non-genetic DNA methylation PCs for DNA methylation.

In order to identify potential molecular mechanisms mediating the GWAS signals, we colocalized the GWAS signals with three molecular QTL datasets, TwinsUK adipose eQTLs, TwinsUK adipose meQTLs, and multi-tissue eQTLs and sQTLs from the GTEx study. We then directly compared the levels of adipose molecular traits (fatty acids, expression, methylation) at colocalized loci. In brief, we found three loci at *SCD*, *FADS*, and 3p25.2 influenced both molecular traits and adipose fatty acids (Table 5; Figure 4). Further colocalization and sensitivity analysis plots are presented in Figures S6, S7, and S8. Direct comparison of adipose DNA methylation, gene expression, and fatty acids by genotypic class are presented in Figures 5 and S9.

**Table 5:** Colocalization of GWAS and adipose eQTL signals

**Adipose fatty acid**	**Chr**	**Lead GWAS SNP**	**Ensembl ID**	**Gene symbol**	**Lead eQTL SNP**	**LD r**^2^	**PP.H4**	**Colocalized SNP**
**Fatty acid**								

SFA	10	rs603424	ENSG00000099194	*SCD*	rs603424	1.00	1.00	rs603424
C16:0 (palmitic acid)	10	rs603424	ENSG00000099194	*SCD*	rs603424	1.00	1.00	rs603424
C18:0 (stearic acid)	10	rs603424	ENSG00000099194	*SCD*	rs603424	1.00	1.00	rs603424
C16:1n-7 (palmitoleic acid)	10	rs603424	ENSG00000099194	*SCD*	rs603424	1.00	1.00	rs603424
C20:4n-6 (arachidonic acid)	11	rs97384	ENSG00000168496	*FEN1*	rs174536	0.76	0.79	rs174601
C20:4n-6 (arachidonic acid)	11	rs97384	ENSG00000149485	*FADS1*	rs4246215	0.68	0.86	rs97384
C20:4n-6 (arachidonic acid)	3	rs1542848	ENSG00000075975	*MKRN2*^a^	rs299628	0.63	0.76	rs1542848

**Fatty acid ratio**								

C16:1n-7/C16:0 (palmitoleic acid/palmitic acid)	10	rs603424	ENSG00000099194	*SCD*	rs603424	1.00	1.00	rs603424
C18:1n-9/C18:0 (oleic acid/stearic acid)	10	rs603424	ENSG00000099194	*SCD*	rs603424	1.00	1.00	rs603424
C20:4n-6/C20:3n-6 (arachidonic acid/dihomo-γ-linolenic acid)	11	rs174544	ENSG00000168496	*FEN1*	rs174536	0.81	0.96	rs174533
C20:4n-6/C20:3n-6 (arachidonic acid/dihomo-γ-linolenic acid)	11	rs174544	ENSG00000134825	*TMEM258*	rs174538	0.67	0.95	rs174544
C20:4n-6/C20:3n-6 (arachidonic acid/dihomo-γ-linolenic acid)	11	rs174544	ENSG00000149485	*FADS1*	rs4246215	0.75	0.95	rs174544

LD r^2^, linkage disequilibrium between GWAS and eQTL lead SNPs; PP.H4, posterior probability of hypothesis 4, both traits are associated and share a single causal variant; Colocalized SNP, the lead colocalized SNP for both GWAS and eQTL studies.

a Secondary signal of the gene *MKRN2*.

At the *SCD* loci, we found evidence of adipose-specific molecular regulation of the associated GWAS signals. We identified strong co-localizations between the *SCD* GWAS signals and the *SCD* adipose primary eQTL (Table 5) and eight nearby adipose CpGs meQTLs (Table S7). In GTEx, *SCD* eQTL co-localizations were presented only in visceral and subcutaneous adipose tissue and a weaker association in breast mammary tissue (which contains adipocytes), supporting adipose-specific regulation at this locus (Table S8). Within the TwinsUK adipose datasets, we observed a direct association between increased levels of *SCD* expression and increased palmitoleic acid, palmitoleic acid/palmitic acid ratio, and oleic acid/stearic acid ratio and decreased levels of palmitic and stearic acids (Table S10). *SCD* adipose expression is highly heritability (h^2^ = 42%), and the lead eQTL/GWAS variant explains ∼10% of the variance of this gene.

In contrast, at the *FADS* cluster, we found evidence of colocalization of the GWAS signals with multiple genes and tissues, suggesting more complex, tissue-shared regulation. While the GWAS signals colocalized with adipose eQTLs of *FADS1*, *FEN1*, and *TMEM258* (Table 5) and eight CpGs meQTLs (Table S7) within the TwinsUK adipose datasets, in GTEx we also found colocalization with eQTLs of multiple genes, including *FADS1*, *FADS2*, *FEN1*, and *TMEM258* across multiple tissues, including adipose subcutaneous, adipose visceral omentum, pancreas, lung, artery tibial, colon transverse, verve tibial, esophagus muscularis, esophagus mucosa, and esophagus gastroesophageal junction (Tables S8 and S9), supporting a contributing role of adipose molecular processes to regulation of these fatty acids within a multi-tissue framework. Within the TwinsUK adipose datasets, we found a significant positive association between the ratio of arachidonic acid/dihomo-γ-linolenic acid and adipose expression of *FADS1* (*p* = 5.5 × 10^−5^) and multiple nearby CpGs (Table S11).

Finally, at the 3p25.2 locus, we found evidence of colocalization of the GWAS signals with eight nearby adipose CpGs meQTLs (Table S7) and the secondary adipose eQTL of *MKRN2* (MIM: 608426) (Table 5). Within GTEx, we identified colocalization with eQTLs of *TSEN2* (MIM: 608753) and *RAF1* (MIM: 164760) across multiple tissues (Tables S8 and S9). Notably, we did not identify a colocalization with *PPARG* (MIM: 601487), a regulator of adipocyte differentiation, and the adipose secondary eQTL of *MKRN2* was not present in the GTEx dataset. The ratio of arachidonic acid/dihomo-γ-linolenic acid was directly associated with adipose methylation of multiple CpG probes (Table S11) but not with adipose expression of any nearby genes.

### Fatty acids and kidney traits shared causal variants

**Figure 6:**
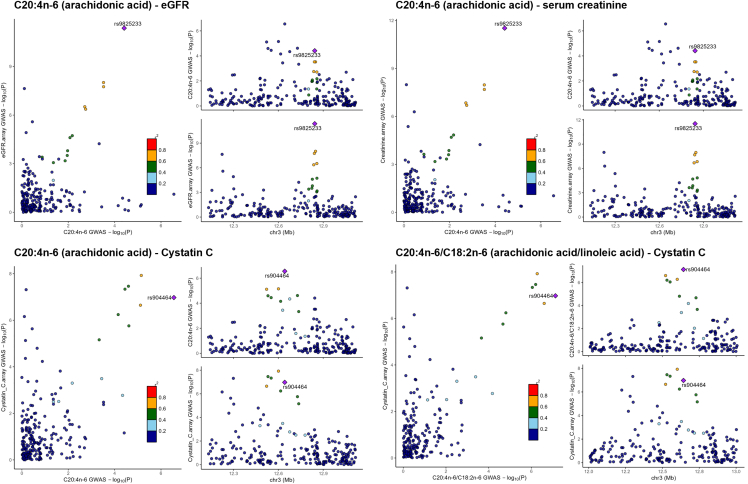
Colocalization of TwinsUK fatty acid GWAS signals and published kidney-trait GWAS signals The labeled variant is the lead variant for both GWAS studies, and other variants are colored according to their LD r^2^ with the lead variant.

In this study, we demonstrated a study-wide significant association between the genetic locus 3p25.2 (near *PPARG*) and adipose tissue levels of arachidonic acid and arachidonic acid/linoleic acid ratio. A recent review has highlighted the implication of PPARγ in critical conditions such as pulmonary arterial hypertension and kidney failure.^55^ eGFR, creatinine, and cystatin C are most widely used renal biomarkers^56^ and available in TwinsUK cohorts. To identify whether the genetic locus 3p25.2 influence both adipose fatty acids and kidney traits, we colocalized Sinnott-Armstrong et al.’s GWAS summary statistics of three kidney traits (eGFR, creatinine, and cystatin C)^45^ with TwinsUK adipose fatty acid GWAS results. We found that eGFR, serum creatinine, and cystatin C shared the single causal variants (rs9825233/rs904464) with arachidonic acid level in adipose tissue, with the posterior probabilities ranging from 0.91 to 0.98. The GWAS signal rs904464 had an effect on both cystatin C level and arachidonic acid/linoleic acid ratio with the posterior probability of 0.99. These suggest that the same genetic locus 3p25.2 influences both adipose n-6 PUFAs and kidney health (Figures 6 and S10).

**Figure 7:**
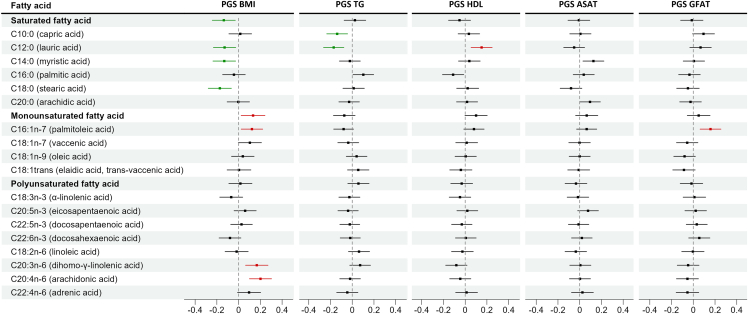
Forest plot of association between polygenic scores and fatty acids in adipose tissue The dots display effect sizes and horizontal lines show their confidence intervals. Significant associations are marked red (positive) and green (negative). BMI, body mass index; TG, total triglycerides; HDL, high-density lipoprotein cholesterol; ASAT, abdominal subcutaneous adipose tissue volumes; GFAT, gluteofemoral adipose tissue volumes.

To further investigate how regulation of fatty acids may correlate with the heritable risks of developing diseases, we utilized PGSs for adiposity traits (visceral, abdominal subcutaneous, and gluteofemoral adipose tissue volumes and waist-to-hip ratio), BMI, lipid biomarkers (HDL, LDL, total triglycerides, and TC), and chronic diseases (T1D and T2D, CAD and hypertension), and fatty acids in adipose tissue (Figure 1; Table S1). The PGSs were first validated by the corresponding trait measures in the full population of TwinsUK (Table S2). We found significant associations between fatty acid levels in adipose tissue and PGSs for BMI, gluteofemoral adipose tissue volumes, HDL, and total triglycerides (Figure 7). More specifically, we observed a negative association of PGS BMI to three SFAs and a positive association of PGS BMI to three unsaturated fatty acids. The PGS for gluteofemoral adipose tissue volumes showed a positive association with palmitoleic acid level. Interestingly, individuals with high levels of lauric acid showed a pattern of elevated PGS HDL and reduced PGS total triglycerides (all adjusted *p* < 0.05; Figure 7; Table S12). In conclusion, polygenic scores of cardio-metabolic traits were associated with adipose tissue fatty acid levels, suggesting genetic risk for these traits also influence fatty acid composition in this key metabolic tissue.

## Discussion

In summary, we characterized the heritability of adipose fatty acids and identified 10 GWAS loci associated with fatty acid levels or fatty acid product-to-precursor ratios in adipose tissue. Through multi-omics colocalization analyses, we prioritized 24 CpGs and five genes mediating the genetic regulation of fatty acids in adipose tissue. Further, using polygenic scores, we linked genetic regulation of adipose fatty acids with biomedical phenotypes.

Fatty acids are important biological molecules, which act as the bedrock for almost all lipid structures and can represent a snapshot of metabolic state. Studies have revealed that circulating fatty acids are associated with T2D, CVD, metabolic syndrome, cognitive function, Alzheimer disease, and inflammatory biomarkers,^11^^,^^57^^,^^58^^,^^59^ but the health impact of different types of fatty acids shows controversial directions. Individual fatty acid contents and fatty acid conversions may be tissue specific, reflecting different lipoprotein classes and tissue-specific modifications. Adipose tissue, which consists almost entirely of triglycerides, is the largest store of fatty acids but also has an endocrine role. However, the regulatory network of fatty acid mechanism in subcutaneous adipose tissue remains unclear. To investigate the genetic regulation of fatty acids in adipose tissue and their links with biomedical traits, we conducted this study.

We revealed a broad range of heritability for individual fatty acids and the ratios of fatty acids, indicating substantial genetic regulation of some adipose fatty acids. The heritability of fatty acids in adipose and serum showed different features in many cases; for example, serum levels of EPA and DHA were greater than those in adipose tissue. Tissue-specific fatty acid heritability may be due to enzymatic activity differences across tissues. The activities of D5D and D6D are higher in serum than in adipose tissue, resulting in higher rate of polyunsaturated fatty acid conversions in serum.^54^^,^^60^ In contrast, some SFAs were not heritable in adipose tissue, possibly because they are primarily obtained from dietary intake, such as coconut oil, palm kernel oil, or bovine milk.^61^

GWASs of heritable fatty acids and ratios identified 10 genetic loci. Two of these loci, at *SCD* and the *FADS* cluster, have been well documented to contain genetic variants that regulate the expression of *SCD* and the *FADS* genes, respectively; these genes encode fatty acid desaturase enzymes (D5D, D6D, and SCD)^62^ that control the conversion rates of fatty acids.^17^ Integration of omic data indicate clear potential molecular mechanisms at these loci; for example, at *SCD*, an increase in the alternative allele frequency of rs603424 (A) is associated with an increase in the adipose methylation levels of nearby probes and a decrease in adipose expression level of *SCD* (Figure 5). Decreasing gene expression of *SCD* will result in less SCD enzyme in a cell, resulting in a slower conversion process from palmitic acid to palmitoleic acid, thereby decreasing the ratio of palmitoleic acid to palmitic acid, consistent with the GWAS associations observed. The eight novel adipose fatty acid-associated loci include the 3p25.2 locus near *PPARG*/*MKRN2*/*TSEN2* genes, which was associated with arachidonic acid and its conversion. The transcription factor peroxisome proliferator-activated receptor (PPAR)-γ2 is expressed specifically for adipose tissue and regulates adipogenic differentiation and lipid storage,^63^ and *MKRN2* regulates the lipid profiling, including TC levels.^64^ Arachidonic acid can generate metabolites including prostaglandins, thromboxane, and leukotrienes, which are associated with the inflammatory damage to the kidney.^65^ By integrating eQTL, meQTL, and renal GWAS data, we provided evidence of the molecular regulation of arachidonic acid (through gene expression and DNA methylation) and its link with renal health. The candidate genes of some novel loci in this study are related to lipid metabolism. *ACSL3* (MIM: 602371) is enriched in lipid droplets and belongs to a family of enzymes that convert free long-chain fatty acids into the substrates for lipid synthesis and β-oxidation, and fatty acyl-CoA esters^66^^,^^67^; *MOGAT1* (MIM: 610268) is highly expressed in adipose tissue^68^ and could be regulated by PPARγ.^69^ Other novel loci also play a vital role in the human body but do not have prior evidence of influencing fatty acid conversion in adipose tissue directly.

Overall adiposity is highly correlated to molecular function and cell-type composition of adipose tissue. When investigating genetic regulation of obesity-related traits, there is an ongoing debate on whether or not to adjust for BMI (or other measures of adiposity) or cell-type composition in analyses. Adjusting for BMI will remove real biological effects that are mediated by BMI and can also potentially introduce false positives via collider bias, as BMI is itself highly heritable.^70^ On the other hand, adjusting for BMI can improve power to detect associations that are not mediated by overall adiposity by removing noise. Given the advantage of both approaches, we chose to not adjust BMI in the main analyses as we wish to capture effects that may be mediated via BMI. We performed sensitivity analyses by re-running the heritability estimates and GWAS analyses including the potential confounders, BMI and/or cell types, as covariates, and we saw similar results across the sensitivity analyses, suggesting that heritabilities and adipose fatty acid-associated loci were still robust when considering other potential confounders. The similarity of results across sensitivity analyses suggests that the genetic regulation of fatty acid levels in adipose tissue is not dependent on the BMI of the individual or the cell-type composition of the sample.

Overall, our results provide insights into the regulation of these key molecules (fatty acids) within a metabolic tissue (adipose tissue). The variants at *SCD*, *FADS1*, and *PPARG* loci have been associated with biomarkers of T2D, cardio-metabolic diseases, and renal diseases; therefore, the knowledge of these adipose fatty acid-associated loci has an impact on understanding these diseases and their molecular etiology. Given the strong association between diet and both adipose fatty acid levels and cardio-metabolic traits, future studies could further dissect whether the genetic regulation of fatty acids could mediate the influence of diet on cardio-metabolic risk.

A limitation of this study is the relatively small sample size, so we are underpowered to detect more genome-wide significant loci of adipose fatty acids, particularly on chromosome X. Given the observed high heritability of adipose fatty acids, and the number of suggestive hits in the GWASs, future studies in a larger population are warranted. Another limitation is that we only integrated adipose tissue of female twins from the UK, therefore follow-up studies including both sexes, other populations with different diets, socio-economic conditions, and living under different geographic and climate conditions are needed to establish the generality of our results to other population. Integration with other molecular layers, such as proteomics and microbiomics, would provide complementary information on how the genome alters fatty acid metabolism in adipose tissue.

In summary, this study identifies heritable adipose fatty acids and their conversions and provides a map of genetic regulation of adipose fatty acids (ratios) where gene expression and DNA methylation act as mediators. We highlight the links between adipose fatty acids and renal and cardio-metabolic health, providing potential pharmaceutical targets for metabolic diseases.

## Data and code availability

TwinsUK fatty acid GWAS datasets of 18 individual fatty acids (adjusted for age, BMI, and family relatedness) can be accessed in the Knowledge Portal of Accelerating Medicines Partnership Common Metabolic Diseases Consortium (AMP CMD Consortium) (https://hugeamp.org/dinspector.html?dataset=Small2023_FattyAcids_EU). Full GWAS summary statistics of all tested fatty acids are available within the CMD Knowledge Portal, allowing incorporation into phenome-wide association study (PheWAS) of other traits. TwinsUK fatty acid GWAS datasets for main analysis and sensitivity analysis are deposited in Zenodo (https://doi.org/10.5281/zenodo.15294670 and https://doi.org/10.5281/zenodo.15508762). TwinsUK RNA-seq data are deposited in the European Genome-phenome Archive (EGA) under accession EGAS00001000805. TwinsUK methylation data are deposited in https://www.ebi.ac.uk/arrayexpress/experiments/E-MTAB-1866/. Access to TwinsUK individual-level data can be obtained by application to the TwinsUK data access committee. For access information and how to apply, please refer to the website (https://twinsuk.ac.uk/researchers/access-data-and-samples/request-access/).

## Acknowledgments

This study was supported by an Accelerating Medicine Partnership Common Metabolic Disease award to K.S.S. and J.T.B. K.S.S. acknowledges support from the 10.13039/501100000265MRC (MR/Y013891/1). This work was in part supported by JPI HDHL-funded DIMENSION project (administered by the 10.13039/501100000268BBSRC UK, grant no. BB/S020845/1 to J.T.B.). X.Y. is funded by King’s-China Scholarship Council (K-CSC) joint scholarship. M.T. is funded by 10.13039/501100000272National Institute for Health and Care Research (NIHR) and Biomedical Research Centre (BRC). C.M. is supported by the 10.13039/100011721Chronic Disease Research Foundation (CDRF) and by the Italian Ministry of Health - Bando Ricerca Corrente 2023. TwinsUK is funded by the 10.13039/100010269Wellcome Trust, 10.13039/501100000265Medical Research Council, 10.13039/501100012041Versus Arthritis, European Union Horizon 2020, CDRF, Zoe Ltd. and the NIHR Clinical Research Network (CRN), and Biomedical Research Centre based at Guy’s and St Thomas’ NHS Foundation Trust in partnership with King’s College London. The contribution of all participants of TwinsUK is gratefully acknowledged. The authors acknowledge use of the research computing facility at King’s College London, King’s Computational Research, Engineering and Technology Environment (CREATE).

## Author contributions

X.Y. performed the main analyses and wrote the paper. K.S.S. designed and supervised the study. S.V., supervised by J.T.B., performed the follow-up meQTL analysis in TwinsUK. M.B.F. supervised GWAS analysis in TwinsUK. A.L.R. and J.S.E.-S.M. contributed to genotype and gene expression data generation. M.T. retrieved and matched cardio-metabolic phenotypes. M.A.-H., supervised by T.A.B.S., detected fatty acid proportions in adipose tissue in TwinsUK. C.M. contributed to generation and quality control of serum metabolite data. K.S.S. and J.T.B. contributed to supervision and overall curation and processing of the data. All authors reviewed and approved the manuscript.

## Declaration of interests

The authors declare no competing interests.

## Supplemental information

Data S1. Tables S1–S12

Document S2. Article plus supplemental information

## References

[1] Oikonomou E.K., Antoniades C. (2019). The role of adipose tissue in cardiovascular health and disease. Nat. Rev. Cardiol..

[2] Smith U., Kahn B.B. (2016). Adipose tissue regulates insulin sensitivity: role of adipogenesis, de novo lipogenesis and novel lipids. J. Intern. Med..

[3] Haffner S.M. (2007). Abdominal adiposity and cardiometabolic risk: do we have all the answers?. Am. J. Med..

[4] Kershaw E.E., Flier J.S. (2004). Adipose tissue as an endocrine organ. J. Clin. Endocrinol. Metab..

[5] Mallick R., Basak S., Duttaroy A.K. (2021). Fatty acids and evolving roles of their proteins in neurological, cardiovascular disorders and cancers. Prog. Lipid Res..

[6] Tabassum R., Rämö J.T., Ripatti P., Koskela J.T., Kurki M., Karjalainen J., Palta P., Hassan S., Nunez-Fontarnau J., Kiiskinen T.T.J. (2019). Genetic architecture of human plasma lipidome and its link to cardiovascular disease. Nat. Commun..

[7] Long T., Hicks M., Yu H.C., Biggs W.H., Kirkness E.F., Menni C., Zierer J., Small K.S., Mangino M., Messier H. (2017). Whole-genome sequencing identifies common-to-rare variants associated with human blood metabolites. Nat. Genet..

[8] Suhre K., Shin S.-Y., Petersen A.-K., Mohney R.P., Meredith D., Wägele B., Altmaier E., Deloukas P., Erdmann J., CARDIoGRAM (2011). Human metabolic individuality in biomedical and pharmaceutical research. Nature.

[9] Shin S.Y., Fauman E.B., Petersen A.K., Krumsiek J., Santos R., Huang J., Arnold M., Erte I., Forgetta V., Yang T.P. (2014). An atlas of genetic influences on human blood metabolites. Nat. Genet..

[10] Surendran P., Stewart I.D., Au Yeung V.P.W., Pietzner M., Raffler J., Wörheide M.A., Li C., Smith R.F., Wittemans L.B.L., Bomba L. (2022). Rare and common genetic determinants of metabolic individuality and their effects on human health. Nat. Med..

[11] Chen Y., Lu T., Pettersson-Kymmer U., Stewart I.D., Butler-Laporte G., Nakanishi T., Cerani A., Liang K.Y.H., Yoshiji S., Willett J.D.S. (2023). Genomic atlas of the plasma metabolome prioritizes metabolites implicated in human diseases. Nat. Genet..

[12] Zierer J., Jackson M.A., Kastenmüller G., Mangino M., Long T., Telenti A., Mohney R.P., Small K.S., Bell J.T., Steves C.J. (2018). The fecal metabolome as a functional readout of the gut microbiome. Nat. Genet..

[13] Schlosser P., Scherer N., Grundner-Culemann F., Monteiro-Martins S., Haug S., Steinbrenner I., Uluvar B., Wuttke M., Cheng Y., Ekici A.B. (2023). Genetic studies of paired metabolomes reveal enzymatic and transport processes at the interface of plasma and urine. Nat. Genet..

[14] Feofanova E.V., Chen H., Dai Y., Jia P., Grove M.L., Morrison A.C., Qi Q., Daviglus M., Cai J., North K.E. (2020). A Genome-wide Association Study Discovers 46 Loci of the Human Metabolome in the Hispanic Community Health Study/Study of Latinos. Am. J. Hum. Genet..

[15] Nag A., Kurushima Y., Bowyer R.C.E., Wells P.M., Weiss S., Pietzner M., Kocher T., Raffler J., Völker U., Mangino M. (2020). Genome-wide scan identifies novel genetic loci regulating salivary metabolite levels. Hum. Mol. Genet..

[16] Klingel S.L., Valsesia A., Astrup A., Kunesova M., Saris W.H.M., Langin D., Viguerie N., Mutch D.M. (2019). FADS1 genotype is distinguished by human subcutaneous adipose tissue fatty acids, but not inflammatory gene expression. Int. J. Obes..

[17] Marklund M., Morris A.P., Mahajan A., Ingelsson E., Lindgren C.M., Lind L., Risérus U. (2018). Genome-Wide Association Studies of Estimated Fatty Acid Desaturase Activity in Serum and Adipose Tissue in Elderly Individuals: Associations with Insulin Sensitivity. Nutrients.

[18] Verdi S., Abbasian G., Bowyer R.C.E., Lachance G., Yarand D., Christofidou P., Mangino M., Menni C., Bell J.T., Falchi M. (2019). TwinsUK: The UK Adult Twin Registry Update. Twin Res. Hum. Genet..

[19] Christie W.W. (1990).

[20] Alhilal M. (2013).

[21] Lankinen M.A., Stančáková A., Uusitupa M., Ågren J., Pihlajamäki J., Kuusisto J., Schwab U., Laakso M. (2015). Plasma fatty acids as predictors of glycaemia and type 2 diabetes. Diabetologia.

[22] Visscher P.M., Benyamin B., White I. (2004). The use of linear mixed models to estimate variance components from data on twin pairs by maximum likelihood. Twin Res..

[23] Glastonbury C.A., Couto Alves A., El-Sayed Moustafa J.S., Small K.S. (2019). Cell-Type Heterogeneity in Adipose Tissue Is Associated with Complex Traits and Reveals Disease-Relevant Cell-Specific eQTLs. Am. J. Hum. Genet..

[24] Zhou X., Stephens M. (2012). Genome-wide efficient mixed-model analysis for association studies. Nat. Genet..

[25] Shungin D., Winkler T.W., Croteau-Chonka D.C., Ferreira T., Locke A.E., Mägi R., Strawbridge R.J., Pers T.H., Fischer K., Justice A.E. (2015). New genetic loci link adipose and insulin biology to body fat distribution. Nature.

[26] Devlin B., Roeder K. (1999). Genomic control for association studies. Biometrics.

[27] Boughton A.P., Welch R.P., Flickinger M., VandeHaar P., Taliun D., Abecasis G.R., Boehnke M. (2021). LocusZoom.js: Interactive and embeddable visualization of genetic association study results. Bioinformatics.

[28] Buil A., Brown A.A., Lappalainen T., Viñuela A., Davies M.N., Zheng H.F., Richards J.B., Glass D., Small K.S., Durbin R. (2015). Gene-gene and gene-environment interactions detected by transcriptome sequence analysis in twins. Nat. Genet..

[29] Dobin A., Davis C.A., Schlesinger F., Drenkow J., Zaleski C., Jha S., Batut P., Chaisson M., Gingeras T.R. (2013). STAR: ultrafast universal RNA-seq aligner. Bioinformatics.

[30] Raulerson C.K., Ko A., Kidd J.C., Currin K.W., Brotman S.M., Cannon M.E., Wu Y., Spracklen C.N., Jackson A.U., Stringham H.M. (2019). Adipose Tissue Gene Expression Associations Reveal Hundreds of Candidate Genes for Cardiometabolic Traits. Am. J. Hum. Genet..

[31] Glastonbury C.A., Viñuela A., Buil A., Halldorsson G.H., Thorleifsson G., Helgason H., Thorsteinsdottir U., Stefansson K., Dermitzakis E.T., Spector T.D., Small K.S. (2016). Adiposity-Dependent Regulatory Effects on Multi-tissue Transcriptomes. Am. J. Hum. Genet..

[32] Delaneau O., Ongen H., Brown A.A., Fort A., Panousis N.I., Dermitzakis E.T. (2017). A complete tool set for molecular QTL discovery and analysis. Nat. Commun..

[33] Frankish A., Diekhans M., Ferreira A.M., Johnson R., Jungreis I., Loveland J., Mudge J.M., Sisu C., Wright J., Armstrong J. (2019). GENCODE reference annotation for the human and mouse genomes. Nucleic Acids Res..

[34] Robinson M.D., Oshlack A. (2010). A scaling normalization method for differential expression analysis of RNA-seq data. Genome Biol..

[35] Grundberg E., Meduri E., Sandling J.K., Hedman A.K., Keildson S., Buil A., Busche S., Yuan W., Nisbet J., Sekowska M. (2013). Global analysis of DNA methylation variation in adipose tissue from twins reveals links to disease-associated variants in distal regulatory elements. Am. J. Hum. Genet..

[36] Du P., Zhang X., Huang C.C., Jafari N., Kibbe W.A., Hou L., Lin S.M. (2010). Comparison of Beta-value and M-value methods for quantifying methylation levels by microarray analysis. BMC Bioinf..

[37] Christiansen C., Tomlinson M., Eliot M., Nilsson E., Costeira R., Xia Y., Villicaña S., Mompeo O., Wells P., Castillo-Fernandez J. (2022). Adipose methylome integrative-omic analyses reveal genetic and dietary metabolic health drivers and insulin resistance classifiers. Genome Med..

[38] Tsai P.C., Glastonbury C.A., Eliot M.N., Bollepalli S., Yet I., Castillo-Fernandez J.E., Carnero-Montoro E., Hardiman T., Martin T.C., Vickers A. (2018). Smoking induces coordinated DNA methylation and gene expression changes in adipose tissue with consequences for metabolic health. Clin. Epigenetics.

[39] El-Sayed Moustafa J.S., Jackson A.U., Brotman S.M., Guan L., Villicaña S., Roberts A.L., Zito A., Bonnycastle L., Erdos M.R., Narisu N. (2022). ACE2 expression in adipose tissue is associated with cardio-metabolic risk factors and cell type composition-implications for COVID-19. Int. J. Obes..

[40] Brotman S.M., El-Sayed Moustafa J.S., Guan L., Broadaway K.A., Wang D., Jackson A.U., Welch R., Currin K.W., Tomlinson M., Vadlamudi S. (2025). Adipose tissue eQTL meta-analysis highlights the contribution of allelic heterogeneity to gene expression regulation and cardiometabolic traits. Nat. Genet..

[41] GTEx Consortium (2020). The GTEx Consortium atlas of genetic regulatory effects across human tissues. Science.

[42] Min J.L., Hemani G., Hannon E., Dekkers K.F., Castillo-Fernandez J., Luijk R., Carnero-Montoro E., Lawson D.J., Burrows K., Suderman M. (2021). Genomic and phenotypic insights from an atlas of genetic effects on DNA methylation. Nat. Genet..

[43] Giambartolomei C., Vukcevic D., Schadt E.E., Franke L., Hingorani A.D., Wallace C., Plagnol V. (2014). Bayesian Test for Colocalisation between Pairs of Genetic Association Studies Using Summary Statistics. PLoS Genet..

[44] Bates D., Mächler M., Bolker B., Walker S. (2015). Fitting Linear Mixed-Effects Models Using lme4. J. Stat. Software.

[45] Sinnott-Armstrong N., Tanigawa Y., Amar D., Mars N., Benner C., Aguirre M., Venkataraman G.R., Wainberg M., Ollila H.M., Kiiskinen T. (2021). Genetics of 35 blood and urine biomarkers in the UK Biobank. Nat. Genet..

[46] Agrawal S., Wang M., Klarqvist M.D.R., Smith K., Shin J., Dashti H., Diamant N., Choi S.H., Jurgens S.J., Ellinor P.T. (2022). Inherited basis of visceral, abdominal subcutaneous and gluteofemoral fat depots. Nat. Commun..

[47] Pulit S.L., Stoneman C., Morris A.P., Wood A.R., Glastonbury C.A., Tyrrell J., Yengo L., Ferreira T., Marouli E., Ji Y. (2019). Meta-analysis of genome-wide association studies for body fat distribution in 694 649 individuals of European ancestry. Hum. Mol. Genet..

[48] Mansour Aly D., Dwivedi O.P., Prasad R.B., Käräjämäki A., Hjort R., Thangam M., Åkerlund M., Mahajan A., Udler M.S., Florez J.C. (2021). Genome-wide association analyses highlight etiological differences underlying newly defined subtypes of diabetes. Nat. Genet..

[49] Aragam K.G., Jiang T., Goel A., Kanoni S., Wolford B.N., Atri D.S., Weeks E.M., Wang M., Hindy G., Zhou W. (2022). Discovery and systematic characterization of risk variants and genes for coronary artery disease in over a million participants. Nat. Genet..

[50] Zhang H., Zhan J., Jin J., Zhang J., Lu W., Zhao R., Ahearn T.U., Yu Z., O'Connell J., Jiang Y. (2023). A new method for multiancestry polygenic prediction improves performance across diverse populations. Nat. Genet..

[51] Zheng Z., Liu S., Sidorenko J., Yengo L., Turley P., Ani A., Wang R., Nolte I.M., Snieder H., Yang J. (2022). Leveraging functional genomic annotations and genome coverage to improve polygenic prediction of complex traits within and between ancestries. bioRxiv.

[52] Weissbrod O., Kanai M., Shi H., Gazal S., Peyrot W.J., Khera A.V., Okada Y., Martin A.R., Finucane H.K., Price A.L., Biobank Japan Project (2022). Leveraging fine-mapping and multipopulation training data to improve cross-population polygenic risk scores. Nat. Genet..

[53] Craig L.C.A., Thies F., Brittenden J., Kyle J., McNeill G. (2009). Relative validity of fatty acid intakes from an FFQ compared with subcutaneous adipose tissue fatty acids. Proc. Nutr. Soc..

[54] Hodson L., Skeaff C.M., Fielding B.A. (2008). Fatty acid composition of adipose tissue and blood in humans and its use as a biomarker of dietary intake. Prog. Lipid Res..

[55] Kokeny G., Calvier L., Hansmann G. (2021). PPARgamma and TGFbeta-Major Regulators of Metabolism, Inflammation, and Fibrosis in the Lungs and Kidneys. Int. J. Mol. Sci..

[56] Pottel H., Delanaye P., Cavalier E. (2024). Exploring Renal Function Assessment: Creatinine, Cystatin C, and Estimated Glomerular Filtration Rate Focused on the European Kidney Function Consortium Equation. Ann. Lab. Med..

[57] Wang D.C., Sun C.H., Liu L.Y., Sun X.H., Jin X.W., Song W.L., Liu X.Q., Wan X.L. (2012). Serum fatty acid profiles using GC-MS and multivariate statistical analysis: potential biomarkers of Alzheimer's disease. Neurobiol. Aging.

[58] Wang L., Folsom A.R., Eckfeldt J.H. (2003). Plasma fatty acid composition and incidence of coronary heart disease in middle aged adults: the Atherosclerosis Risk in Communities (ARIC) Study. Nutr. Metab. Cardiovasc. Dis..

[59] Zhuang P., Liu X., Li Y., Li H., Zhang L., Wan X., Wu Y., Zhang Y., Jiao J. (2022). Circulating Fatty Acids and Genetic Predisposition to Type 2 Diabetes: Gene-Nutrient Interaction Analysis. Diabetes Care.

[60] Nankam P.A.N., Jaarsveld P.J.V., Chorell E., Smidt M.C.F.d., Adams K., Blüher M., Olsson T., Mendham A.E., Goedecke J.H. (2020). Circulating and Adipose Tissue Fatty Acid Composition in Black South African Women with Obesity: A Cross-Sectional Study. Nutrients.

[61] Seidelin K.N. (1995). Fatty acid composition of adipose tissue in humans. Implications for the dietary fat-serum cholesterol-CHD issue. Prog. Lipid Res..

[62] Lattka E., Illig T., Koletzko B., Heinrich J. (2010). Genetic variants of the FADS1 FADS2 gene cluster as related to essential fatty acid metabolism. Curr. Opin. Lipidol..

[63] Kotani K., Saiga K., Kurozawa Y., Sakane N., Sano Y., Tabata M. (2007). The peroxisome proliferator-activated receptor gamma2 gene Pro12Ala polymorphism and serum C-reactive protein in general Japanese population. Clin. Chim. Acta.

[64] Ghanbari M., Franco O.H., de Looper H.W.J., Hofman A., Erkeland S.J., Dehghan A. (2015). Genetic Variations in MicroRNA-Binding Sites Affect MicroRNA-Mediated Regulation of Several Genes Associated With Cardio-metabolic Phenotypes. Circ. Cardiovasc. Genet..

[65] Wang T., Fu X., Chen Q., Patra J.K., Wang D., Wang Z., Gai Z. (2019). Arachidonic Acid Metabolism and Kidney Inflammation. Int. J. Mol. Sci..

[66] Soupene E., Kuypers F.A. (2008). Mammalian long-chain acyl-CoA synthetases. Exp. Biol. Med..

[67] Poppelreuther M., Rudolph B., Du C., Großmann R., Becker M., Thiele C., Ehehalt R., Füllekrug J. (2012). The N-terminal region of acyl-CoA synthetase 3 is essential for both the localization on lipid droplets and the function in fatty acid uptake. J. Lipid Res..

[68] Sankella S., Garg A., Agarwal A.K. (2016). Characterization of the Mouse and Human Monoacylglycerol O-Acyltransferase 1 (Mogat1) Promoter in Human Kidney Proximal Tubule and Rat Liver Cells. PLoS One.

[69] Yu J.H., Lee Y.J., Kim H.J., Choi H., Choi Y., Seok J.W., Kim J.W. (2015). Monoacylglycerol O-acyltransferase 1 is regulated by peroxisome proliferator-activated receptor gamma in human hepatocytes and increases lipid accumulation. Biochem. Biophys. Res. Commun..

[70] Day F.R., Loh P.R., Scott R.A., Ong K.K., Perry J.R.B. (2016). A Robust Example of Collider Bias in a Genetic Association Study. Am. J. Hum. Genet..

