# Supplementary material for: Genetic regulation of fatty acid content in adipose tissue
Source: Am J Hum Genet. 2026 Jan 14;113(2):291–308. doi: 10.1016/j.ajhg.2025.12.008 (PMC13087407; doi:10.1016/j.ajhg.2025.12.008)
Supplement: Document S1. Figures S1–S10 [file mmc1.pdf]

The American Journal of Human Genetics, Volume *113*

Supplemental information

Genetic regulation of fatty acid content in adipose tissue

Xinyu Yan, Amy L. Roberts, Julia S. El-Sayed Moustafa, Sergio Villicaña, Maryam Al-Hilal, Max Tomlinson, Cristina Menni, Thomas A.B. Sanders, Maxim B. Freidin, Jordana T. Bell, and Kerrin S. Small

Supplemental Material and Methods

Polygenic score

To construct polygenic scores (PGSs) for adiposity, BMI, waist-to-hip ratio, lipid biomarkers and chronic diseases, we searched terms on the PGS Catalog or GWAS performed in large cohorts of European ancestry. We calculated 13 PGSs: abdominal subcutaneous adipose tissue volumes adjusted for BMI (ASATadjBMI), visceral adipose tissue volumes adjusted for BMI (VATadjBMI), gluteofemoral adipose tissue volumes adjusted for BMI (GFATadjBMI), BMI, waist-to-hip ratio adjusted for BMI (WHRadjBMI), type 1 diabetes (T1D), T2D, coronary artery disease (CAD), hypertension, high-density lipoproteins cholesterol (HDL), low-density lipoproteins cholesterol (LDL), log-transformed total triglycerides (TG) and total cholesterol (TC) [1-7] (Table S1). This study analyzed the following 12 cardio-metabolic risk indicators: Percentage of fat in android region (AF), percentage of fat in the largest visceral fat region (VF), percentage of fat in gynoid region (GF), BMI, the ratio of percentage of fat in the largest visceral fat region to percentage of fat in gynoid region (VRGF), systolic blood pressure, diastolic blood pressure, HDL, LDL, TG, TC, and Atherosclerotic Cardiovascular Disease (ASCVD) risk score. AF, VAF and GF were measured via dual-energy X-ray absorptiometry scans (Hologic QDR; Hologic, Inc.) and VRGF was calculated afterward. During the clinical visit, the height and weight of the participants were measured by trained nurses following the harmonized protocols to calculate BMI. Blood pressure was measured using Marshall mb02, Omron Mx3 or Omron HEM713C Digital Blood Pressure Monitor with the participant sitting for at

least three minutes by a trained nurse. Hypertension is considered to be from 140/90 mmHg or more (age < 80) or 150/90 mmHg or more (age ≥ 80) or self-reported anti-hypertension drug use. The lipid profiles and glycemic indicators levels were measured after an overnight fasting period according to standard procedures (Biochemistry Department, King's College Hospital, London and Affinity Biomarker Labs, London, United Kingdom). The 10-year ASCVD risk score is an algorithm utilized to estimate the cardiovascular risk of the participant. Detailed description of clinical indicators has been previously reported [8-10].

To validate the PGSs in TwinsUK, we hypothesized that the highest decile of PGS tends to reside in the highest decile trait or disease group, and vice versa. We only selected one twin in each pair and each trait was residualized against the covariates included in the original paper. We found that participants at the tails of the distribution for BMI and log triglycerides were enriched in extreme PGSs. For example, participants in the top 10% of the BMI distribution were nearly five times as likely to have a BMI polygenic score in the top 10% of the distribution (41.8% vs. 8.5%; OR = 4.92; 95% CI: 3.56 - 6.79). Conversely, individuals with less than the 10th percentile of BMI were over three times as likely to have a BMI polygenic score less than the 10th percentile (32.9% vs. 9.1%; OR = 3.61; 95% CI: 2.59 - 5.05) (Figure S1). The decile with the highest CVD PGS had a 1.73 (1.12 - 2.67) times higher risk of CAD.

To test the differences of morphological profiles on the tail ends of PGSs of diseases, individuals belonging to top 10% and bottom 10% of PGS distribution were categorized into two levels, then independent t-test was conducted. PGSs of CVD, T2D and

hypertension revealed significant differences between disease and non-disease groups (t = -3.094, P = 0.002; t = -4.235, $P = 2.4 \times 10^{-5}$; t = -5.378, $P = 8.3 \times 10^{-8}$). The adherence of PGSs to corresponding traits were predicted using the continuous PGS variable among all female twins adjusted for relatedness and covariates applied in the source paper. Overall, PGSs showed broadly strong associations with corresponding traits in TwinsUK cohort (Beta = 0.028 - 0.397, FDR < 0.05, Table S2).

Supplemental References

1. Agrawal, S., Wang, M., Klarqvist, M.D.R., Smith, K., Shin, J., Dashti, H., Diamant, N., Choi, S.H., Jurgens, S.J., Ellinor, P.T., et al. (2022). Inherited basis of visceral, abdominal subcutaneous and gluteofemoral fat depots. Nat Commun *13*, 3771. 10.1038/s41467-022-30931-2.
2. Pulit, S.L., Stoneman, C., Morris, A.P., Wood, A.R., Glastonbury, C.A., Tyrrell, J., Yengo, L., Ferreira, T., Marouli, E., Ji, Y., et al. (2019). Meta-analysis of genome-wide association studies for body fat distribution in 694 649 individuals of European ancestry. Hum Mol Genet *28*, 166-174. 10.1093/hmg/ddy327.
3. Mansour Aly, D., Dwivedi, O.P., Prasad, R.B., Karajamaki, A., Hjort, R., Thangam, M., Akerlund, M., Mahajan, A., Udler, M.S., Florez, J.C., et al. (2021). Genome-wide association analyses highlight etiological differences underlying newly defined subtypes of diabetes. Nat Genet *53*, 1534-1542. 10.1038/s41588-021-00948-2.
4. Aragam, K.G., Jiang, T., Goel, A., Kanoni, S., Wolford, B.N., Atri, D.S., Weeks, E.M., Wang, M., Hindy, G., Zhou, W., et al. (2022). Discovery and systematic characterization of risk variants and genes for coronary artery disease in over a million participants. Nat Genet *54*, 1803-1815. 10.1038/s41588-022-01233-6.
5. Zhang, H., Zhan, J., Jin, J., Zhang, J., Lu, W., Zhao, R., Ahearn, T.U., Yu, Z., O'Connell, J., Jiang, Y., et al. (2023). A new method for multiancestry polygenic prediction improves performance across diverse populations. Nat Genet *55*, 1757-1768. 10.1038/s41588-023-01501-z.
6. Zheng, Z., Liu, S., Sidorenko, J., Yengo, L., Turley, P., Ani, A., Wang, R., Nolte, I.M., Snieder, H., Yang, J., et al. (2022). Leveraging functional genomic annotations and genome coverage to improve polygenic prediction of complex traits within and between ancestries. bioRxiv. 10.1101/2022.10.12.510418.
7. Weissbrod, O., Kanai, M., Shi, H., Gazal, S., Peyrot, W.J., Khera, A.V., Okada, Y., Biobank Japan, P., Martin, A.R., Finucane, H.K., and Price, A.L. (2022). Leveraging

fine-mapping and multipopulation training data to improve cross-population polygenic risk scores. Nat Genet *54*, 450-458. 10.1038/s41588-022-01036-9.

8. Menni, C., Migaud, M., Glastonbury, C.A., Beaumont, M., Nikolaou, A., Small, K.S., Brosnan, M.J., Mohney, R.P., Spector, T.D., and Valdes, A.M. (2016). Metabolomic profiling to dissect the role of visceral fat in cardiometabolic health. Obesity (Silver Spring) *24*, 1380-1388. 10.1002/oby.21488.

9. Menni, C., Gudelj, I., Macdonald-Dunlop, E., Mangino, M., Zierer, J., Besic, E., Joshi, P.K., Trbojevic-Akmacic, I., Chowienczyk, P.J., Spector, T.D., et al. (2018). Glycosylation Profile of Immunoglobulin G Is Cross-Sectionally Associated With Cardiovascular Disease Risk Score and Subclinical Atherosclerosis in Two Independent Cohorts. Circ Res *122*, 1555-1564. 10.1161/CIRCRESAHA.117.312174.

10. Jennings, A., MacGregor, A., Welch, A., Chowienczyk, P., Spector, T., and Cassidy, A. (2015). Amino Acid Intakes Are Inversely Associated with Arterial Stiffness and Central Blood Pressure in Women. J Nutr *145*, 2130-2138. 10.3945/jn.115.214700.

Supplemental Data

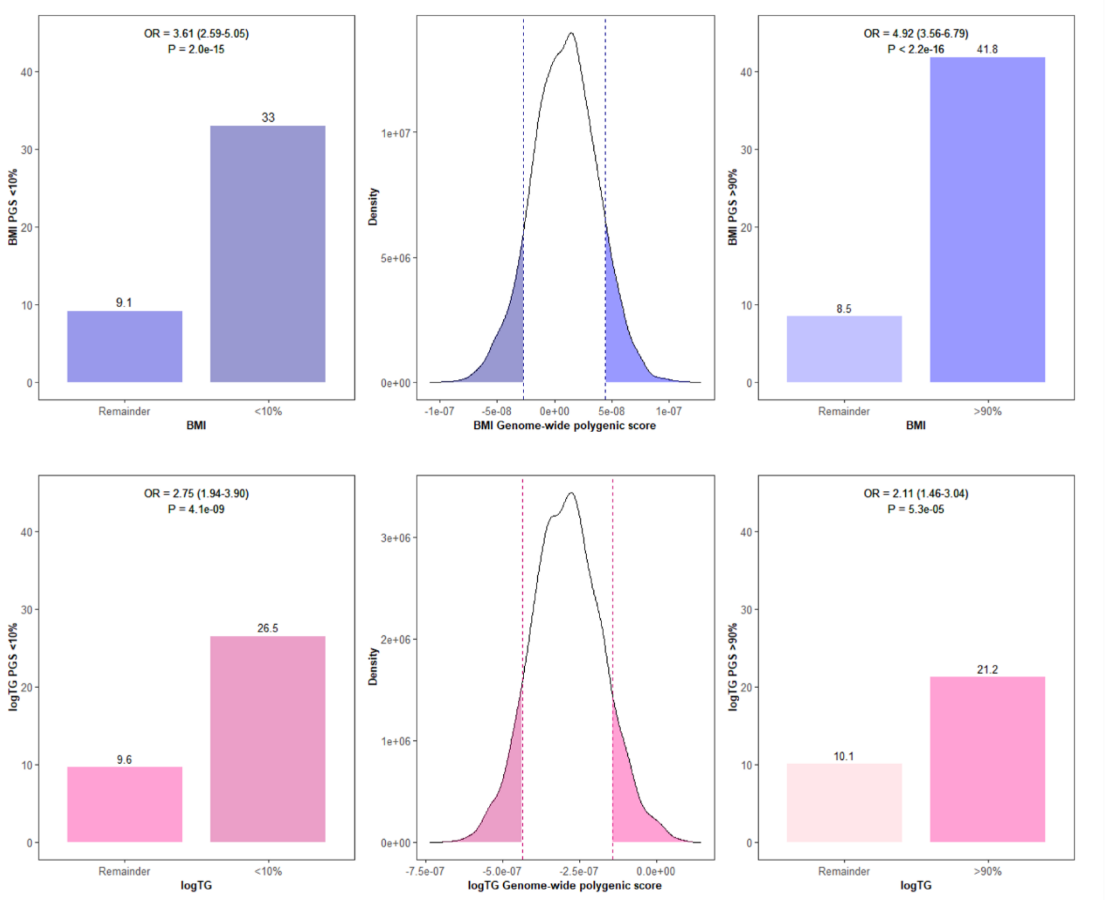

Figure S1. Enrichment of BMI and log triglycerides genome-wide polygenic scores in tails of the distribution.

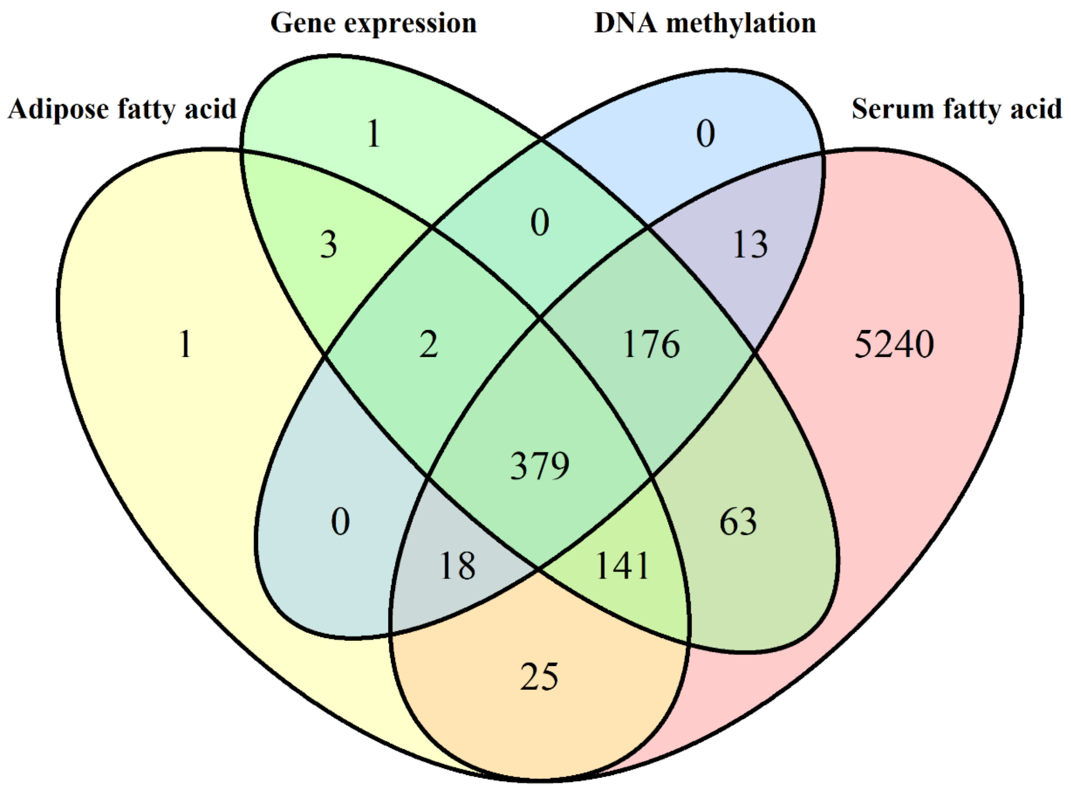

	Adipose fatty acid	Gene expression	DNA methylation	Serum fatty acid
**Adipose fatty acid**	569	--	--	--
**Gene expression**	525	765		--
**DNA methylation**	399	557	588	--
**Serum fatty acid**	563	759	586	6055

Figure S2. Venn plot and grid of individuals present in each dataset.

To note, among 563 twins with both serum fatty acid and adipose fatty acid data, 448 twins' blood and biopsy samples were collected in the same clinical visit.

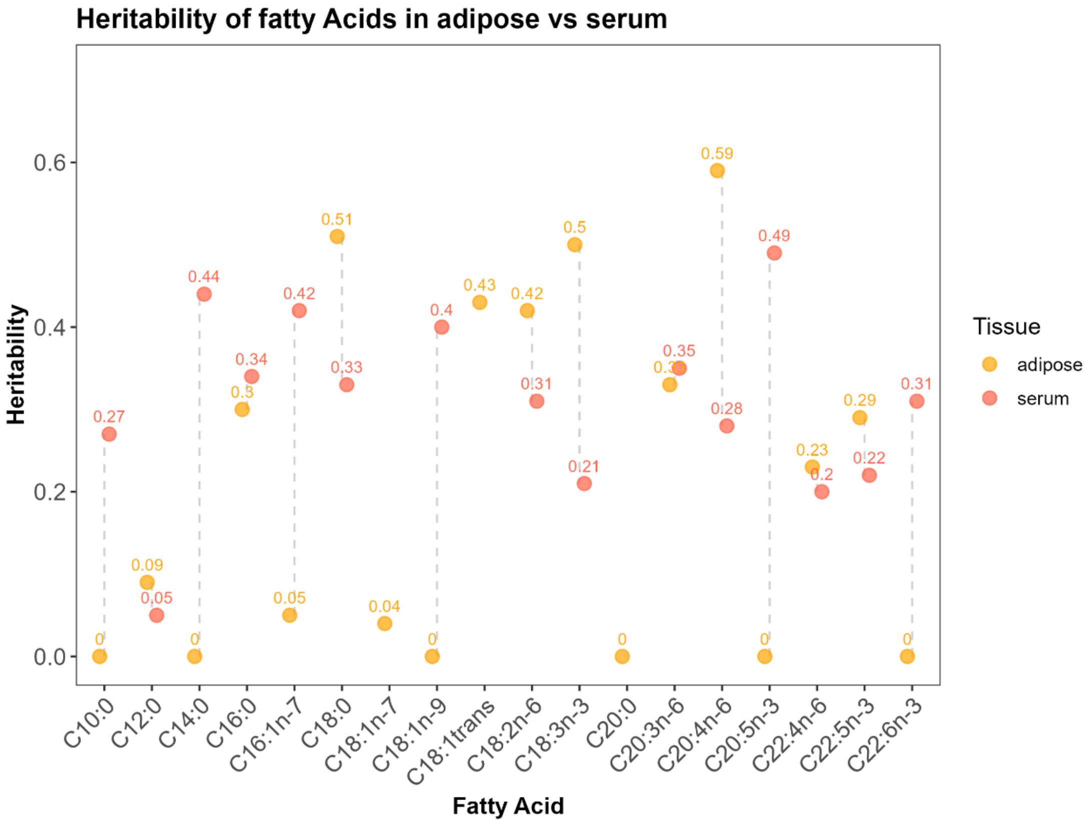

Figure S3. Heritability of individual fatty acids across tissues (adipose vs serum).

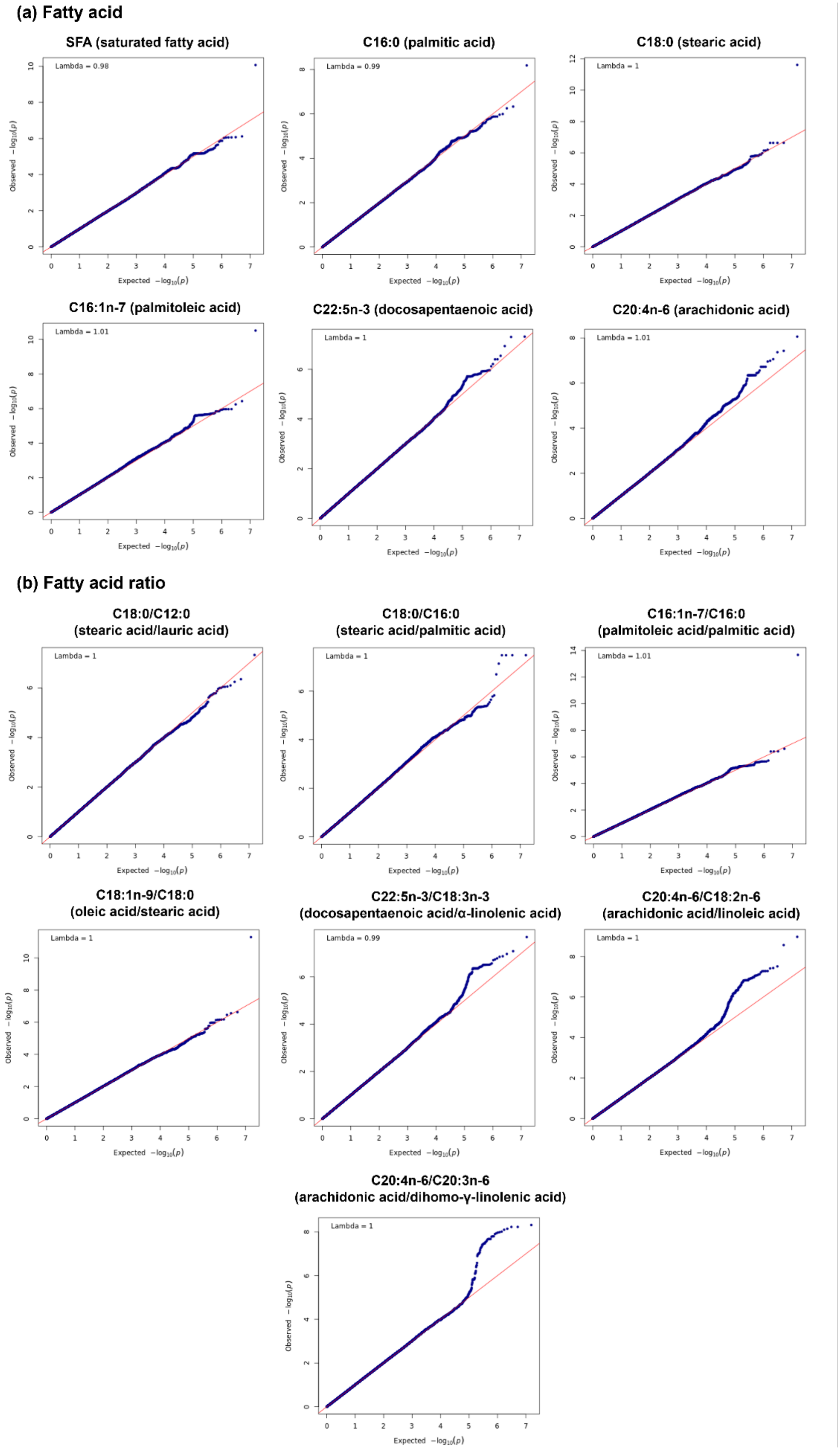

Figure S4. QQ plots of GWASs on fatty acids in adipose tissue.

QQ plots illustrate the findings of genome-wide association study for adipose fatty acids. The red diagonal line demarcates null hypothesis. Genomic inflation was close to one, suggesting no evidence of inflation.

(a) Fatty acid

SFA (saturated fatty acid)

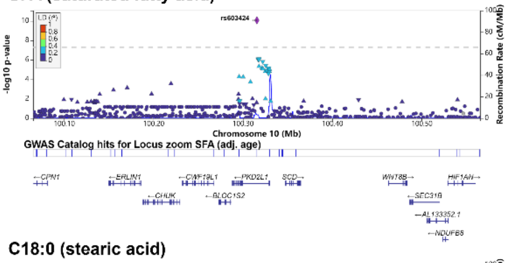

C16:0 (palmitic acid)

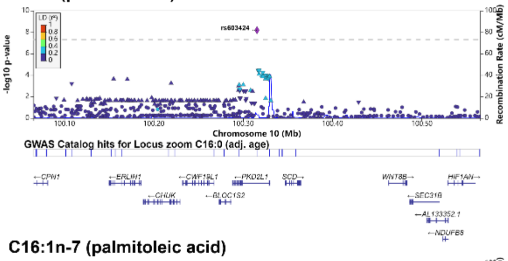

C18:0 (stearic acid)

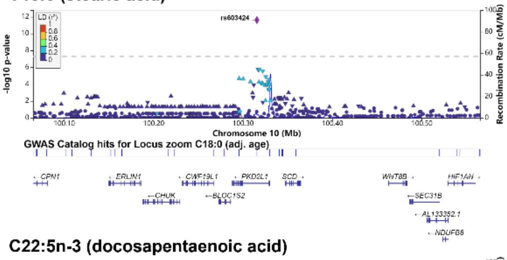

C16:1n-7 (palmitoleic acid)

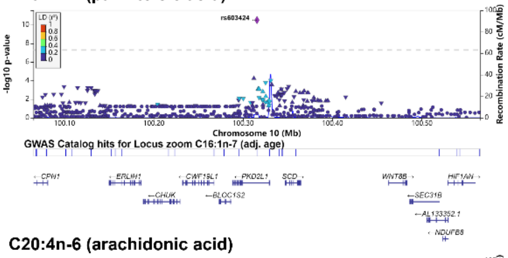

C22:5n-3 (docosapentaenoic acid)

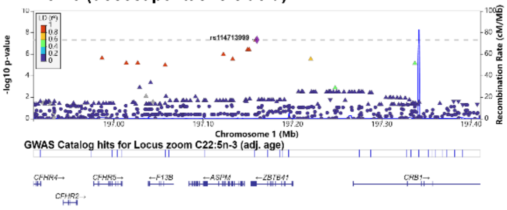

C20:4n-6 (arachidonic acid)

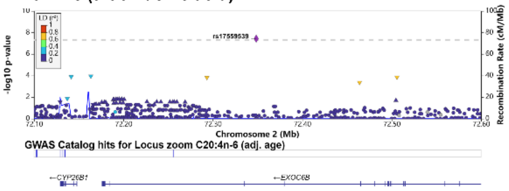

C20:4n-6 (arachidonic acid)

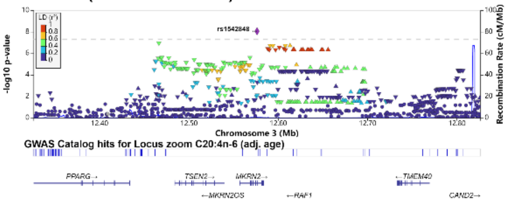

C20:4n-6 (arachidonic acid)

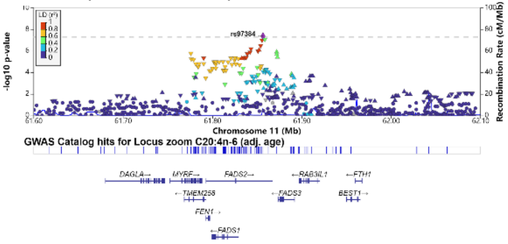

C20:4n-6 (arachidonic acid) – condition on the lead SNP

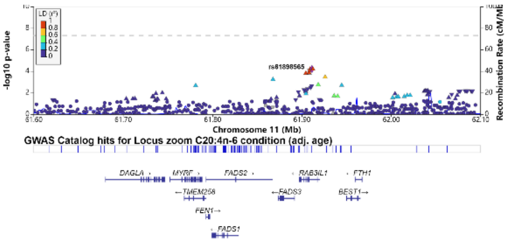

(b) Fatty acid ratio

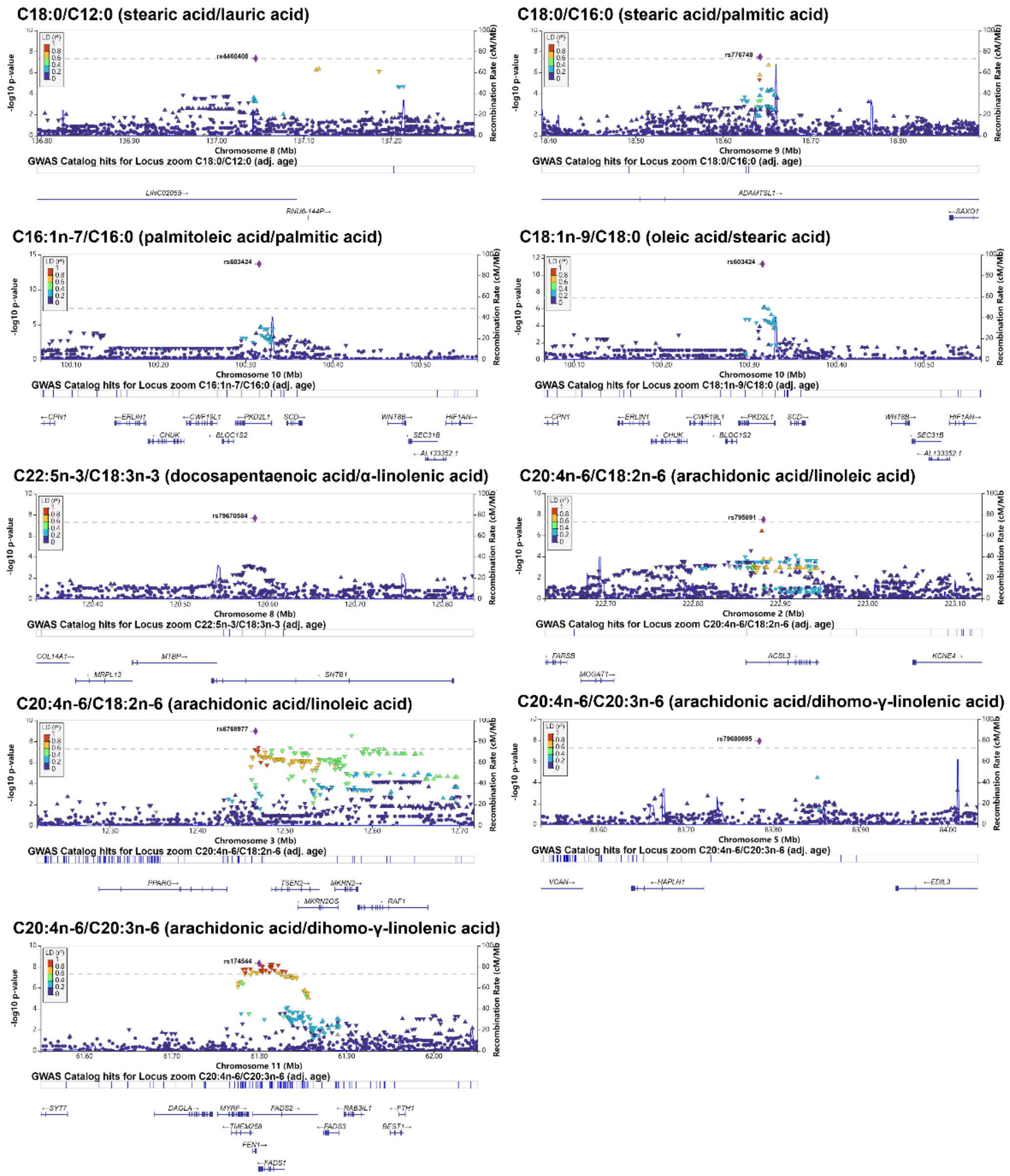

Figure S5. Locus zoom plots of GWASs on fatty acids in adipose tissue.

Locus zoom plots illustrate genome-wide association results of adipose fatty acids at significant loci.

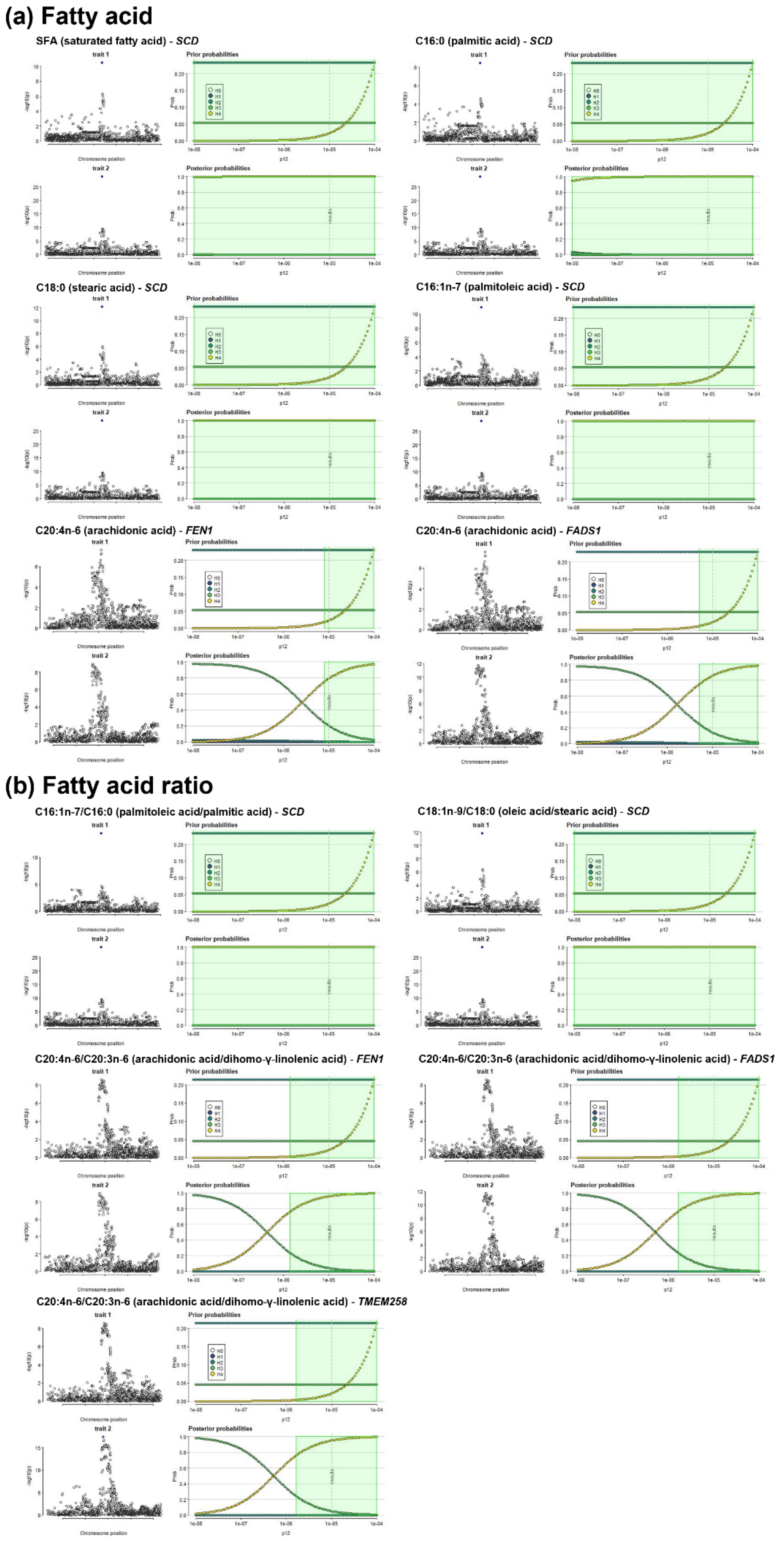
(a) Fatty acid
SFA (saturated fatty acid) - SCD
C16:0 (palmitic acid) - SCD
C18:0 (stearic acid) - SCD
C16:1n-7 (palmitoleic acid) - SCD
C20:4n-6 (arachidonic acid) - FEN1
C20:4n-6 (arachidonic acid) - FADS1
(b) Fatty acid ratio
C16:1n-7/C16:0 (palmitoleic acid/palmitic acid) - SCD
C18:1n-9/C18:0 (oleic acid/stearic acid) - SCD
C20:4n-6/C20:3n-6 (arachidonic acid/dihomo-γ-linolenic acid) - FEN1
C20:4n-6/C20:3n-6 (arachidonic acid/dihomo-γ-linolenic acid) - FADS1
C20:4n-6/C20:3n-6 (arachidonic acid/dihomo-γ-linolenic acid) - TMEM258
trait 1
trait 2
Prior probabilities
Posterior probabilities
Chromosome position
p12
H0
H1
H2
H3
H4

Figure S6. Sensitivity analysis of colocalization between GWAS and eQTL signals.

On the left the input data are presented, with shading to indicate the posterior probabilities (PP) that a SNP is causal if hypothesis 4 (H4) is true. On the right the green region shows the region, the set of values of p12, for which PP.H4>0.75.

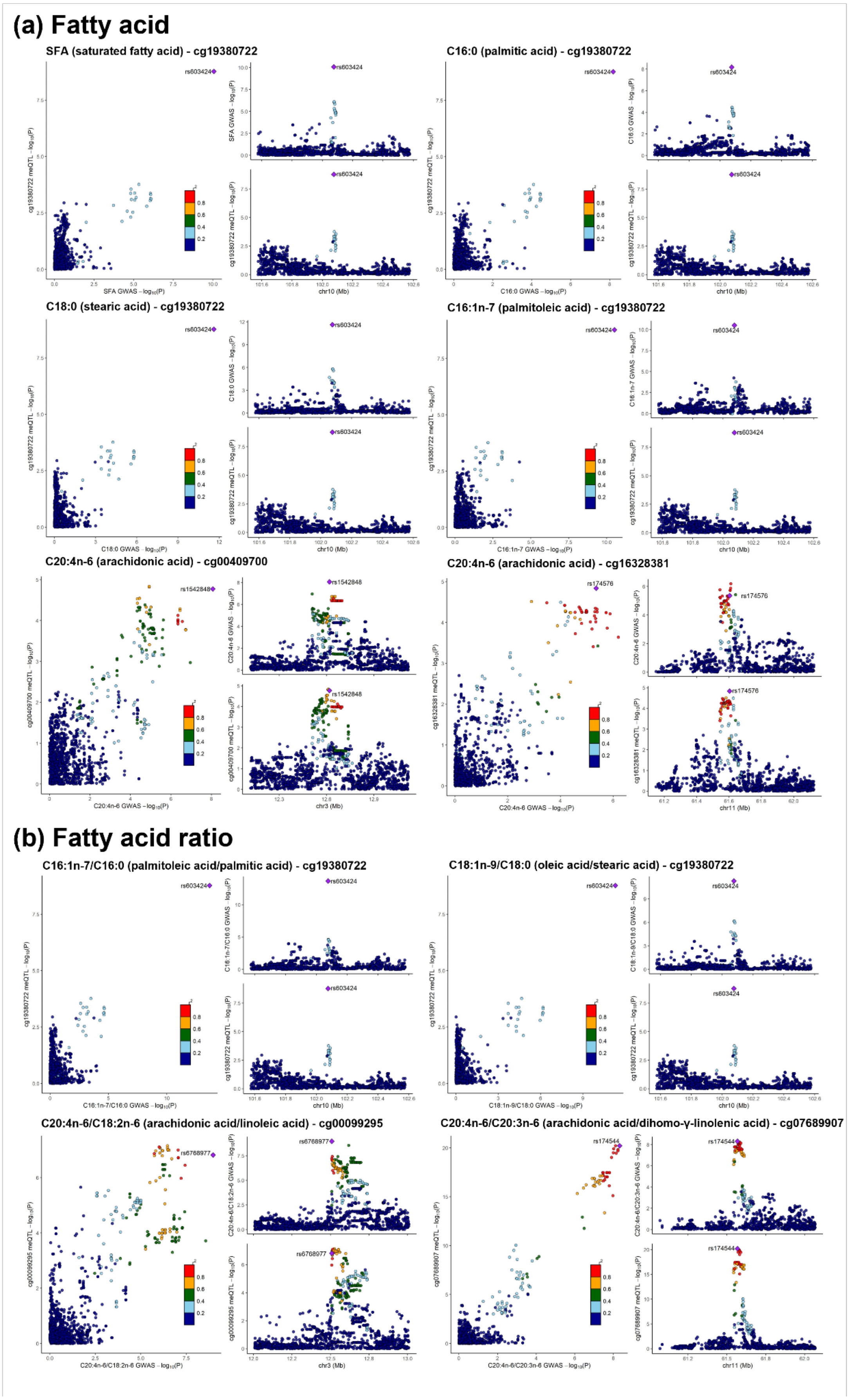

Figure S7. Colocalization of GWAS and meQTL signals.

The labeled SNP is the lead colocalized SNP for both GWAS and meQTL studies, and other SNPs are colored according to their LD r^2 with the lead SNP.

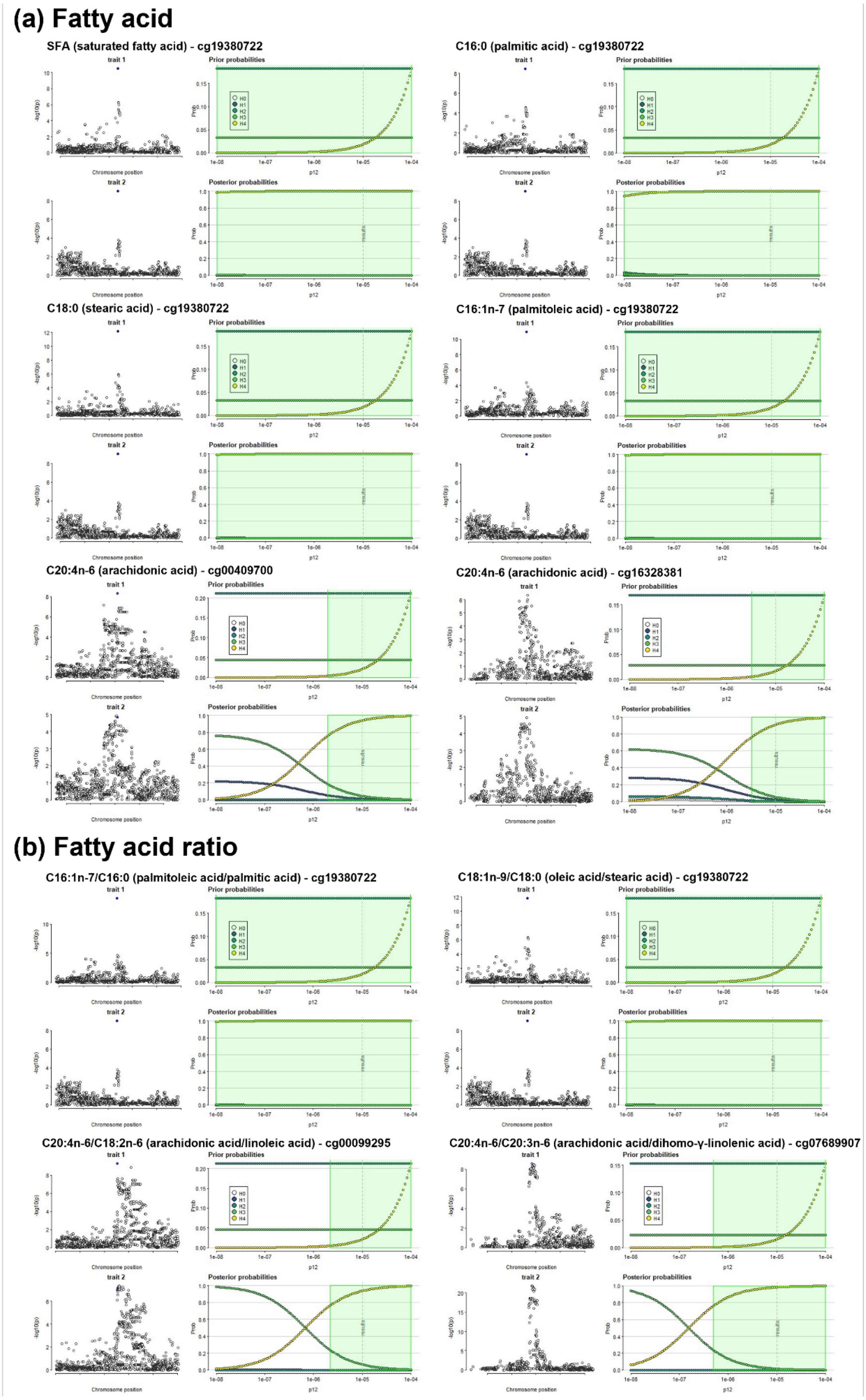

Figure S8. Sensitivity analysis of colocalization between GWAS and meQTL signals.

On the left the input data are presented, with shading to indicate the posterior probabilities (PP) that a SNP is causal if hypothesis 4 (H4) is true. On the right the green region shows the region, the set of values of p12, for which PP.H4>0.75.

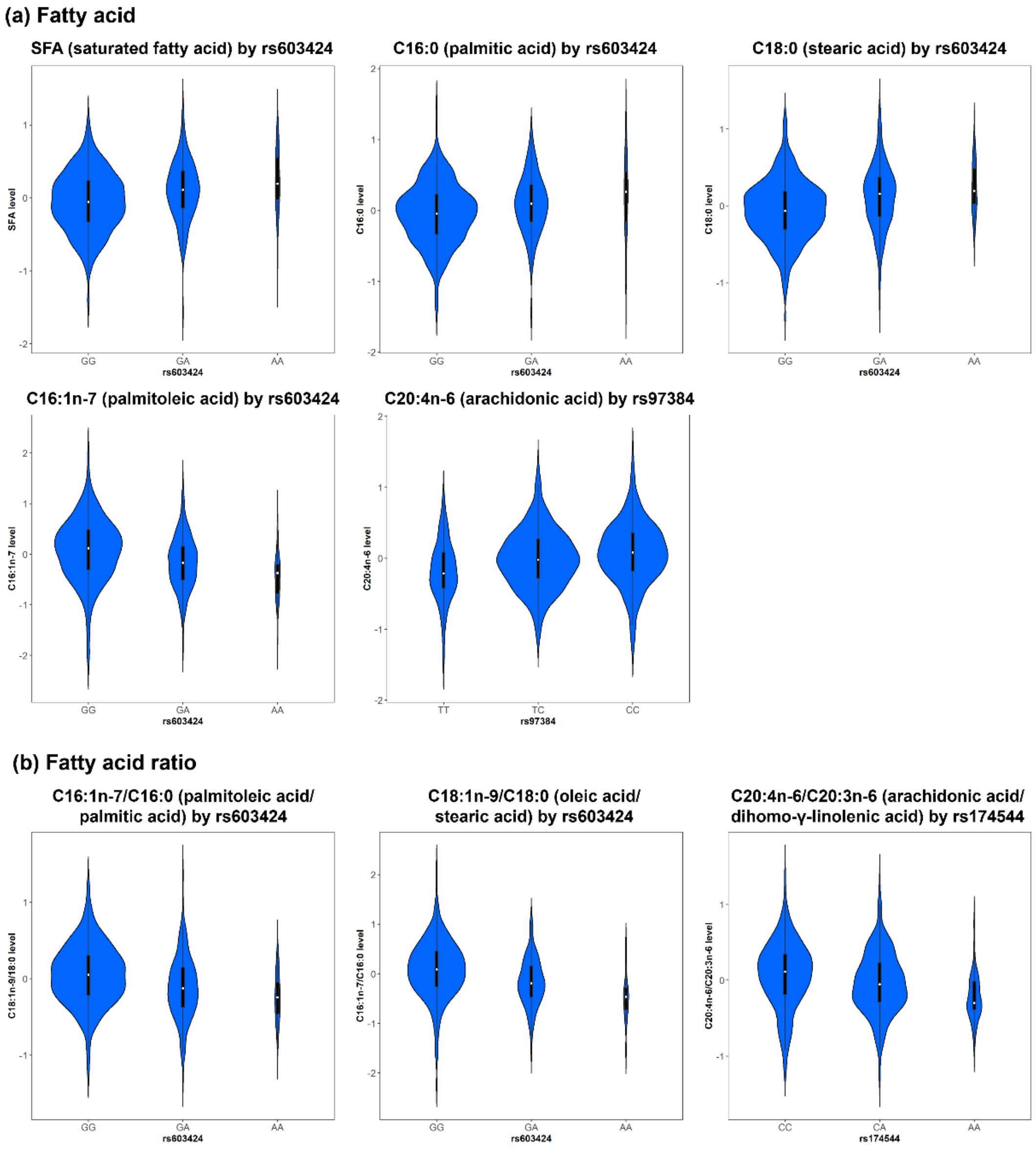

Figure S9. Violin plots of fatty acids by genotypic class.

The plots show the adipose fatty acid levels stratified by genotypes The effect of these SNP on fatty acids are potentially mediated through gene expression and/or DNA methylation.

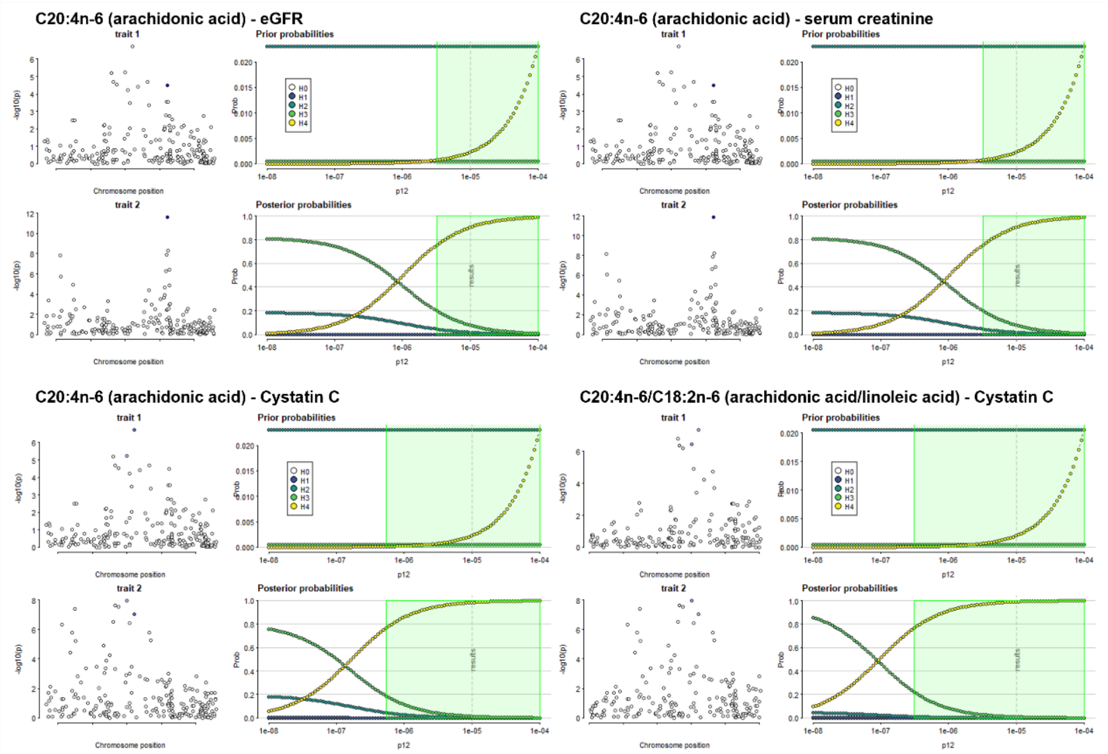

Figure S10. Sensitivity analysis of colocalization between TwinsUK fatty acid GWAS and published kidney trait GWAS signals.

On the left the input data are presented, with shading to indicate the posterior probabilities (PP) that a SNP is causal if hypothesis 4 (H4) is true. On the right the green region shows the region, the set of values of p12, for which PP.H4>0.75.
